# Population Structure Shapes Copy Number Variation in Malaria Parasites

**DOI:** 10.1093/molbev/msv282

**Published:** 2015-11-26

**Authors:** Ian H. Cheeseman, Becky Miller, John C. Tan, Asako Tan, Shalini Nair, Standwell C. Nkhoma, Marcos De Donato, Hectorina Rodulfo, Arjen Dondorp, Oralee H. Branch, Lastenia Ruiz Mesia, Paul Newton, Mayfong Mayxay, Alfred Amambua-Ngwa, David J. Conway, François Nosten, Michael T. Ferdig, Tim J. C. Anderson

**Affiliations:** ^1^Department of Genetics, Texas Biomedical Research Institute, San Antonio, TX; ^2^The Eck Institute for Global Health, Department of Biological Sciences, University of Notre Dame; ^3^Malawi-Liverpool-Wellcome Trust Clinical Research Programme, University of Malawi College of Medicine, Blantyre, Malawi; ^4^Lab. Genetica Molecular, IIBCAUDO, Universidad De Oriente, Cumana, Venezuela; ^5^Mahidol-Oxford Tropical Medicine Research Unit, Faculty of Tropical Medicine, Mahidol University, Bangkok, Thailand; ^6^Centre for Tropical Medicine and Global Health, Nuffield Department of Medicine, Churchill Hospital, University of Oxford, Oxford, United Kingdom; ^7^Division of Parasitology, Department of Microbiology, New York University School of Medicine; ^8^Laboratorio De Investigaciones De Productos Naturales Y Antiparasitarios, Universidad Nacional De La Amazonia Peruana, Iquitos, Peru; ^9^Lao-Oxford-Mahosot Hospital-Wellcome Trust Research Unit (LOMWRU), Microbiology Laboratory, Mahosot Hospital, Vientiane, Lao PDR; ^10^Faculty of Postgraduate Studies, University of Health Sciences, Vientiane, Lao PDR; ^11^Medical Research Council Unit, Fajara, Banjul, The Gambia; ^12^Department of Pathogen Molecular Biology, London School of Hygiene and Tropical Medicine, London, United Kingdom; ^13^Shoklo Malaria Research Unit, Mahidol-Oxford Tropical Medicine Research Unit, Faculty of Tropical Medicine, Mahidol University, Mae Sot, Thailand

**Keywords:** copy number variation, population genetics, parasitology.

## Abstract

If copy number variants (CNVs) are predominantly deleterious, we would expect them to be more efficiently purged from populations with a large effective population size (*N*_e_) than from populations with a small *N*_e_. Malaria parasites (*Plasmodium falciparum*) provide an excellent organism to examine this prediction, because this protozoan shows a broad spectrum of population structures within a single species, with large, stable, outbred populations in Africa, small unstable inbred populations in South America and with intermediate population characteristics in South East Asia. We characterized 122 single-clone parasites, without prior laboratory culture, from malaria-infected patients in seven countries in Africa, South East Asia and South America using a high-density single-nucleotide polymorphism/CNV microarray. We scored 134 high-confidence CNVs across the parasite exome, including 33 deletions and 102 amplifications, which ranged in size from <500 bp to 59 kb, as well as 10,107 flanking, biallelic single-nucleotide polymorphisms. Overall, CNVs were rare, small, and skewed toward low frequency variants, consistent with the deleterious model. Relative to African and South East Asian populations, CNVs were significantly more common in South America, showed significantly less skew in allele frequencies, and were significantly larger. On this background of low frequency CNV, we also identified several high-frequency CNVs under putative positive selection using an *F*_ST_ outlier analysis. These included known adaptive CNVs containing *rh2b* and *pfmdr1*, and several other CNVs (e.g., *DNA helicase* and three conserved proteins) that require further investigation. Our data are consistent with a significant impact of genetic structure on CNV burden in an important human pathogen.

## Introduction

Copy number variation (CNV) is widespread in eukaryotic genomes, in taxa ranging from yeast (Liti et al. 2009) to humans (Mills et al. 2011) and multiple organisms in between (Emerson et al. 2008; Brown et al. 2012). The selective forces determining the distribution of CNVs within populations are poorly understood. Several clear examples of the beneficial nature of CNVs exist. For instance, amplification of the CCL3L1 gene lowers risk of HIV progression (Gonzalez et al. 2005), and amylase copy number correlates with dietary starch levels in both humans (Perry et al. 2007) and domesticated dogs (Axelsson et al. 2013). However, there is accumulating evidence that CNV may be generally deleterious and subject to purifying selection. This notion has several lines of support; 1) the size spectrum of CNVs is skewed toward smaller variants, consistent with the expectation that longer variants tend to have a greater impact on fitness. 2) The allele frequency spectrum of CNVs is generally skewed toward rare variants (Conrad et al. 2010; Mills et al. 2011; Sjodin and Jakobsson 2012). Additionally, coding regions of the genome show lower levels of CNV than noncoding regions (Conrad et al. 2010) because structural variation within coding sequence has a greater potential to be deleterious. This may result from disruption of gene function due to truncations, frameshifts, duplications, or deletions.

If CNVs are typically deleterious, we expect that the burden of CNVs will scale inversely with the effective population size (*N_e_*), because the strength of purifying selection is strongest in large populations (Ohta 1992). Under this model, populations with large *N_e_* will contain a lower burden of CNVs than populations with small *N_e_*, because large populations can purge deleterious mutations more effectively than small populations. This has been suggested as an explanation for the differences in CNV burden between great ape species (Gazave et al. 2011; Sudmant et al. 2013): western chimpanzees show low nucleotide diversity consistent with a population bottleneck (Prado-Martinez et al. 2013) but also show significantly higher levels of both segregating and fixed deletions than other great ape lineages. Supporting evidence also comes from the comparison of three model eukaryotes (*Saccharomyces cerevisiae*, *Drosophila melanogaster* and *Caenorhabditis elegans*) for which estimated *N_e_* are 3.3 × 10^7^, 1.15 × 10^6^^,^ and 8 × 10^4^^,^ respectively, and for which CNV mutation rates have been directly measured by mutation accumulation experiments and calculated bioinformatically from sequence data by measuring the age distribution of gene duplicates (Katju and Bergthorsson 2013). The bioinformatic approach provided estimates 36,000-fold lower than predicted from mutation accumulation experiments for yeast suggesting strong selection against new duplications, but the discrepancy is much lower for *D. melanogaster* (660-fold) and *C. elegans* (340-fold) which have lower *N_e_*. Both great ape and model organism comparisons rely on comparisons between species so are potentially confounded by other differences between these species. Malaria parasites provide a useful system for directly investigating the relationship between genetic parameters of a population (i.e., recombination rate, inbreeding rate, and *N_e_*) and CNV, because this protozoan shows a broad spectrum of population structures within a single species (Anderson et al. 2000; Manske et al. 2012). These range from small, unstable, inbred populations with low recombination rates in South America, to large, outbred populations with very high recombination rates in Africa (Anderson et al. 2000; Joy et al. 2003; Neafsey et al. 2008; Manske et al. 2012). For example, there is a 10–24-fold difference in *N_e_* (Anderson et al. 2000; Joy et al. 2003) and >250-fold difference in the population scaled recombination rate (Mu et al. 2005) between African and South American parasite populations. These differences have been repeatedly confirmed using mitochondrial DNA and single-nucleotide polymorphism (SNP) and microsatellite analyses of nuclear polymorphisms (Anderson et al. 2000; Joy et al. 2003; Mu et al. 2005; Neafsey et al. 2008). These diversity-based measures parallel differences in census population size in contemporary parasite populations, with an estimated 122 million *P. falciparum* cases in Africa, 3 million in SE Asia, and 0.4 million in South America (World Health Organization 2014). The central goal of this project was to compare the impact of *N_e_* (and associated population parameters) on copy number dynamics in a single species, rather than relying on cross species comparisons.

There have been several previous surveys of CNV in *P. falciparum* (Carret et al. 2005; Kidgell et al. 2006; Ribacke et al. 2007; Jiang et al. 2008; Cheeseman et al. 2009; Mackinnon et al. 2009). However, these previous studies have two limitations. First, these studies have used parasite isolates that were grown in cell culture media in the laboratory, to ensure sufficient DNA for analysis. This is not ideal because CNVs are known to emerge extremely rapidly during laboratory selection. For instance, large chromosomal truncations have been frequently observed following initiation of parasite cultures (Biggs et al. 1989; Shirley et al. 1990; Kemp et al. 1992) or between isogenic clones (Carret et al. 2005) and amplification of the *Rh1* gene is observed in 30–40% of laboratory lines though has yet to be observed in field isolates (Nery et al. 2006; Jennings et al. 2007; Mackinnon et al. 2009; Nair et al. 2010). Second, the numbers of parasites examined in previous studies were modest, ranging from two (Carret et al. 2005) to 16 (Cheeseman et al. 2009), precluding rigorous comparisons between parasite populations. Consequently, another goal of this work was to describe the CNV landscape in a global sample of natural *P. falciparum* population obtained directly from the blood of patients to avoid bias resulting from adaptation to laboratory culture.

Prior genome-wide investigations of CNV in the *P. falciparum* genome have suggested that a large proportion of the parasite genome (∼5%) exhibits CNV (Carret et al. 2005; Kidgell et al. 2006; Ribacke et al. 2007; Jiang et al. 2008; Cheeseman et al. 2009; Mackinnon et al. 2009). There has been considerable success in linking CNVs detected in these studies to clinically relevant phenotypes. Notably, detection of gene amplification at *GTP-cyclohydrolase* (Kidgell et al. 2006), a key enzyme in the folate biosynthesis pathway, prompted further characterization of this mutation in field lines (Nair et al. 2008) and through experimental manipulation in the laboratory (Heinberg et al. 2013). These studies suggest that this CNV contributes to the genetic robustness of anti-folate resistance evolution (Kumpornsin et al. 2014). CNVs are involved in other clinically relevant phenotypic traits including mefloquine resistance (Cowman et al. 1994), erythrocyte invasion (Triglia et al. 2005), and cytoadhence/gametocytogenesis (Biggs et al. 1989; Shirley et al. 1990; Kemp et al. 1992) and affect gene expression both locally and distally within the genome (Gonzales et al. 2008; Mackinnon et al. 2009). The breakpoints of CNV regions are preferentially located in A/T-rich regions or homopolymeric tracts (Nair et al. 2007; Guler et al. 2013). Given that the *P. falciparum* genome is strongly AT biased (85% AT (Gardner et al. 2002), that homopolymeric AT tracts are extremely common, and that over 10^11^ parasites are found within infected patients, CNVs are predicted to be a major source of adaptive polymorphism (Nair et al. 2007; Guler et al. 2013). This supposition is strongly supported by laboratory selection experiments with *P. falciparum*. Numerous studies, using selection with different antimalarial drugs, reveal amplification in specific genome regions containing target resistance genes (Thaithong et al. 2001; Singh and Rosenthal 2004; Dharia et al. 2009; Eastman et al. 2011) and this approach is now routinely used to identify genome regions targeted by new drugs (Flannery et al. 2013). While CNV clearly occurs commonly in the laboratory, with duplication rates estimated at approximately 10^−7^ per genome per 48-h asexual cycle for two individual loci (Preechapornkul et al. 2009; Guler et al. 2013), there is a clear need to understand how the spread of beneficial CNVs shapes the malaria parasite genome. A third goal of this study was to identify CNVs that play a role in adaptive evolution of parasites in natural parasite populations.

We examined 122 uncultured, geographically dispersed, malaria parasite isolates containing a single predominant clone using a custom SNP/CGH array (Tan et al. 2011), identifying 134 high-confidence CNVs within the exome and genotyping 10,107 flanking, biallelic SNPs with a global minor allele frequency ≥1%. We used the data to 1) compare CNV distribution and size from across the spectrum of population structures observed in *P. falciparum*, 2) to jointly examine both CNV and SNP variation allowing comparison of population genetics at these different variants, and 3) to determine geographical variation in CNVs to identify variants that may be adaptive and functionally important.

## Results

### Parasite Samples

We excluded *P. falciparum*-infected blood samples containing multiple genotype infections (supplementary table S1, Supplementary Material online), because these complicate scoring of SNPs and CNV and preclude construction of parasite haplotypes. In low-transmission locations, where self-fertilization of parasites commonly occurs, genetically indistinguishable parasites are frequently found in different patients (Anderson et al. 2000, 2010; Echeverry et al. 2013). We included a single representative of each multilocus genotype identified from preliminary genotyping data to avoid oversampling identical or very closely related parasites. Hence, the final set of 157 parasite samples included in the study comprised genetically unique infections containing a single predominant genotype. The preliminary genotyping data illustrate the differences in genetic structure between populations (Anderson et al. 2000). We see high levels of multiple genotype infections and no identical multilocus genotypes in African sites. In contrast in South American locations, there is very little multiple-clone infection, and only 13 independent genotypes were found among the 81 blood samples examined. These trends are consistent with previous analysis of global population structure in *P. falciparum* (Anderson et al. 2000). Inference of global and local population structure is supported by principal component analysis of 10,107 SNPs typed in each of the 122 samples used in the final analysis. The continent and country of origin of each isolate is well captured, with African, South East Asian, and South American isolates forming distinct clusters (supplementary fig. S1, Supplementary Material online).

### Robust Determination of Copy Number Variation

**Fig. 1.**
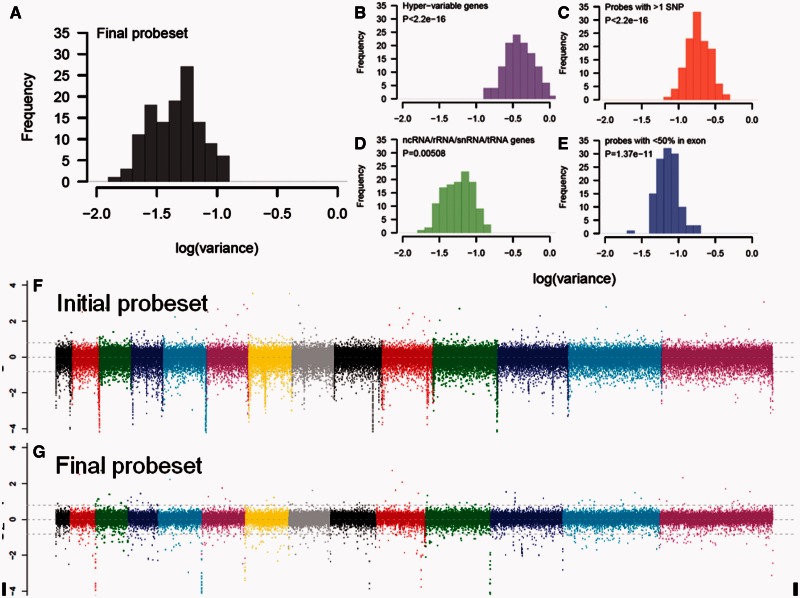
**Generating a high-confidence probe set for robust CNV estimation.** Distribution of probe level variance (on a logarithmic scale) for the final probe set (*A*). The final probe set did not include probes in hypervariable genes (*B*), ncRNA species (*C*), probes with >1 SNP (*D*), and nonexonic probes (*E*), because these showed extremely high variance. The *P* values on each histogram show difference in log(variance) for these particular probe groups relative to other probes within the dataset. Probe level hybridization of a single strain (CAM 11) before (*F*) and after (*G*) removal of probes with high variance. The dotted lines indicate a doubling/halving of signal. Removal of probes showing high variance reduces threshold and improves sensitivity for calling CNVs in the final probe set.

The *P. falciparum* genome, which is > 80% AT-rich and includes nonexonic intervals entirely devoid of C/G residues (Gardner et al. 2002), provides a challenge for CGH-based detection of CNV. We took several steps to ensure the reliability of data analyzed. The SNPs genotyped on the array had previously been subject to extensive validation and yield genotypes with >95% accuracy (Tan et al. 2011; Manske et al. 2012). Before we scored CNV at a population scale, we examined the reliability of all 355,803 CGH probes on our custom array. We were concerned that unreliable probes could either limit our ability to detect CNV due to high background signal or would show an unreasonably high level of false-positive calls. We hybridized 157 samples to the array, excluded 35 which failed our initial quality control thresholds (supplementary fig. S2, Supplementary Material online) and examined variance in probe intensity across the remaining 122 samples. Probes in hypervariable multigene families (*var*/*rifin*/*stevor*) as well as clusters of tRNAs, noncoding RNA species, and ribosomal RNAs showed significantly increased variance in probe intensity across samples compared to genome-wide estimates (fig. 1). While it is likely these gene families harbor abundant CNV, these results suggest we cannot confidently genotype these regions using our array technology. In addition, probes which fell outside exonic regions (<50% of the probe within an exon) or which contained previously described SNPs within their sequence showed significant increase in variance (fig. 1*A–E*). We therefore excluded probes in these categories.

We performed linear regression of probe intensity against both probe length and AT content to correct for residual inconsistencies in probe variance. The decrease in background signal following our quality filtering is demonstrated in figure 1*F* and *G* and resulted in a mean decrease in variance of probe intensity of 24.3% (range 0.75–53.5%). Using this final probe set of 188,081 probes, we segmented the genome into regions of common intensity using CGHseg. This probe set covers 5,148 genes, accounting for 11.6 Mb of the *P. falciparum* genome.

### Reproducibility and Quality Control

To identify appropriate thresholds for determining segments that exhibit CNV, we sequenced four isolates from this study using the Illumina HiSeq and Genome Analyzer II platforms. Previous studies on both humans (Mills et al. 2011) and malaria parasites (Sepulveda et al. 2013) have demonstrated that calling CNV from sequence data using single methods often poorly captures the full extent of variation in a genome. We called CNVs using three algorithms based on either read depth (CNVnator (Abyzov et al. 2011) and FREEC (Boeva et al. 2012)) or read pair distance (BreakDancer (Chen et al. 2009)). We only considered regions retained on the array following our initial quality filtering. By calling segments with ≥8 probes and log_2_ ratio hybridization signal >3 SD below the mean signal (deletions) or > 2.5 SD above the mean (amplifications) as CNVs, 95.7% (114/119) of the CNVs detected on the array overlap with CNVs detected by one or more method in the sequence data (supplementary fig. S3, Supplementary Material online). By varying the threshold used to call CNVs, we empirically determined these thresholds to be optimal. As the malaria parasite is haploid deletion results in a complete removal of sequence compared to an approximate doubling of signal for an amplification. As a result of this effect, the threshold for amplification and deletion detection is not symmetrical. Finally, to assess the reproducibility of our approach, we examined 11 parasites for which a total of 24 hybridizations were conducted (2–4 repeat hybridizations per parasite). Using the thresholds determined above, 74.5% of duplications and 88.6% of deletions (83.7% total) were shared between replicates. Given our high confidence in CNV calling, this likely reflects the false-negative rate in our data.

### Distribution of CNV within the Parasite Genome

The 122 parasite isolates (of 157 hybridized to our array) that passed our quality control criteria (supplementary fig. S2, Supplementary Material online) are described in supplementary table S1, Supplementary Material online. The global sample covers the range of malaria transmission intensities from low (South America: SAM) to intermediate (South East Asia: SEA) to high (Africa: AFR) with 2–3 countries represented from each continent.

We robustly scored 134 CNVs within these samples, including 33 deletions and 102 amplifications, which encompass 306 partial (*n* = 171) or complete (*n* = 135) genes (supplementary fig. S4 and table S2, Supplementary Material online). This constitutes 6% of the 5,148 genes targeted by our final probe set. Deletions ranged in size from 346 bp to 57.9 kb (median = 1,587.5 bp) and spanned up to 10 genes, while amplifications measured from 334 bp to 59.3 kb (median = 1,085.5 bp) and spanned up to 18 genes. Ninety-seven CNVs (17 deletions and 80 amplifications) were biallelic, while 38 CNVs (18 deletions and 20 amplifications) showed clear evidence for multiple alleles (from 3 to 11). For example, at the CNV containing the multidrug resistance locus (chr 5), there are six different amplicons ranging from 10.6 to 26.9 kb and containing from three to six genes, consistent with previous work using real-time polymerase chain reaction (Nair et al. 2007). The number of CNVs per chromosome was strongly correlated with chromosome length (*r^2^* = 0.88; supplementary fig. S4*B*, Supplementary Material online).

### Comparison of CNV between Continents

**Fig. 2.**
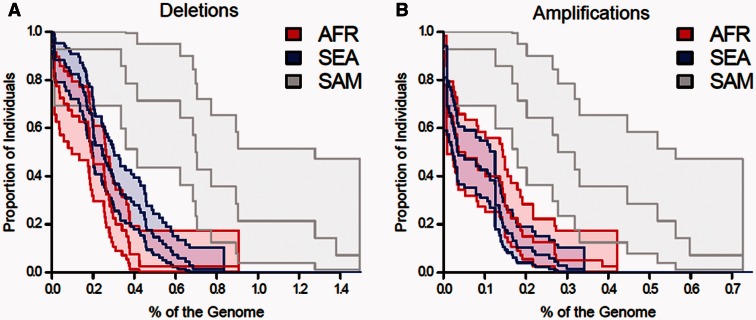
**CNV burden differs between parasite populations from different continents.** Survival curves compare the relationship between CNV burden and proportion of parasites affected for both amplifications (*A*) and deletions (*B*). For each population, central line shows the mean, while the outer lines represent 95% CI. There is a significant excess of CNV in South American populations for both amplifications and deletions.

The burden of CNVs harbored by a genome varied substantially at a continental scale. We measured the burden of CNV in each continental population using two approaches. First, we used survival curves (Sudmant et al. 2013). These incorporate information on CNV size and allele frequency for population comparisons, so fully utilize the data. The heaviest burden of deletions was observed in SAM, followed by SEA, and then AFR. The differences in CNV burden between SEA and AFR populations were not significant, but both of these populations show significantly lower levels of deletions (fig. 2*A*) than SAM populations (AFR vs. SAM: *P* = 7.3 × 10^−9^*:* SEA vs. SAM*, P* = 1.9 × 10^−8^, log-rank test). Continental comparisons of gene amplifications revealed similar patterns (fig. 2*B*), with highly significant increase in amplified genome regions in SAM, relative to AFR (AFR vs. SAM: *P* = 2.5 × 10^−5^, log-rank test) and SEA (SEA vs. SAM: *P* = 1.5 × 10^−6^, log-rank test).

**Fig. 3.**
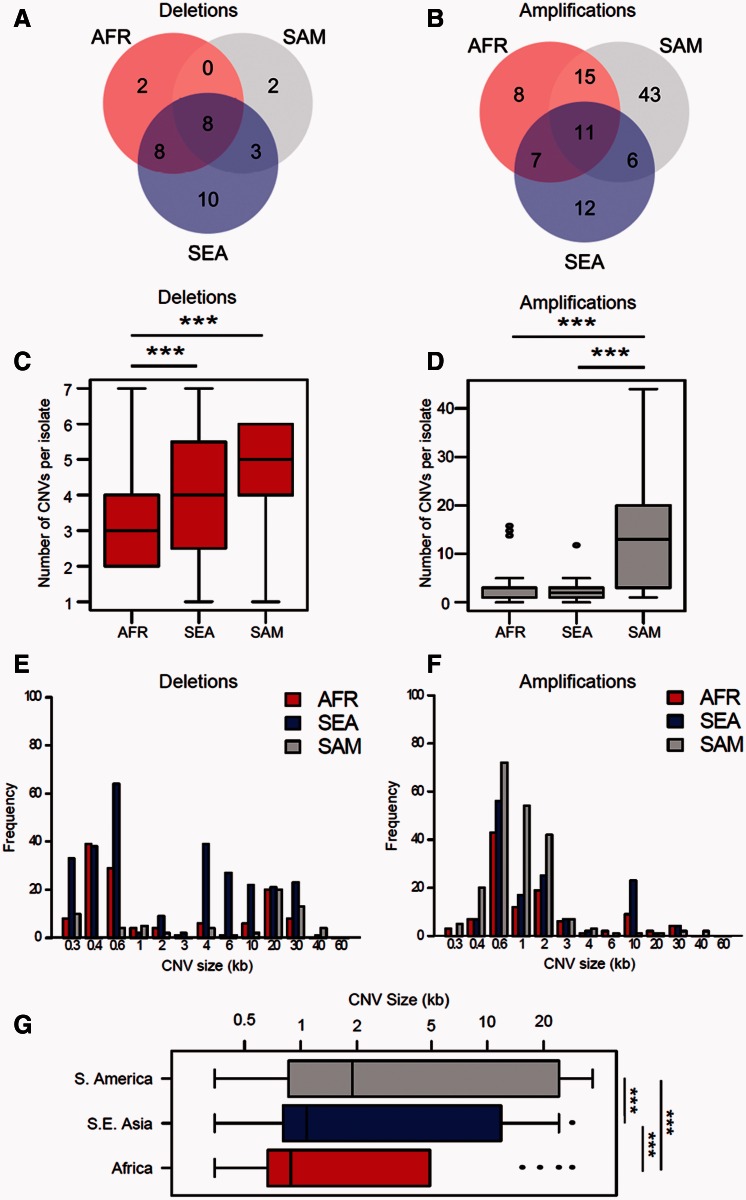
**CNV abundance and size contributes to continental trends in CNV burden.** (*A*, *B*). Numbers of CNVs observed. CNVs that are shared between continents contain at least one gene in common but may differ in size and have independent origins (fig. 9). (*C*, *D*) Plots showing CNV burden per isolate reveal significant differences in the numbers of both deletions and amplifications between continents. (*E*, *F*). Size of CNVs per isolate. There are significant differences in the mean size of CNVs, but the patterns observed differ for deletions and amplifications. Frequency distributions of CNVs stratified by size are shown in figures 5 and 9. (*G*) Size of 19 CNVs common to all three continents. *P < 0.05, **P < 0.01, ***P < 0.001.

The differences in CNV burden revealed by the survival analysis stem from underlying differences in the number, size and allele frequency distribution of CNVs. The number of deletions (fig. 3*A*) and amplifications (fig. 3*B*) show strong differences between locations. These raw numbers are difficult to interpret because sample sizes differ between continents. However, examining mean numbers of CNVs per sample confirmed a significantly lower number of deletions per sample in AFR (fig. 3*C*, mean = 3.15, SD = 1.10) than in SEA (mean = 4.13, SD = 1.76, *P* = 1.9 × 10^−3^, *W* = 1,839.5, Wilcoxon rank sum [WRS] test) or SAM (mean = 4.64, SD = 1.50, *P* = 6.3 × 10^−4^, *W* = 111, WRS test) and that the numbers of amplifications in SAM (fig. 3*D*; mean = 15.00, SD = 13.51) significantly exceeded those observed in both AFR (mean = 2.70, SD = 3.76, *P* = 5.3 × 10^−5^, *W* = 80, WRS test) and SEA (mean = 2.09, SD = 1.66, *P* = 7.4 × 10^−6^, *W* = 122, WRS test).

SAM populations also contained deletions which were significantly longer than either SEA populations (*P* = 2.6 × 10^−6^, *D* = 0.32, Kolmogorov–Smirnov [KS] test) or AFR populations (*P* = 1.3 × 10^−6^, *D* = 0.36, KS test). Similarly, SEA populations harbor larger deletions than AFR populations (*P* = 0.03, *D* = 0.13, KS test, fig. 3*E*). The trend is reversed for amplifications (fig. 3*F*) with SAM populations harboring significantly smaller CNVs than either AFR (*P* = 0.007, *D* = 0.20, KS test) or SEA (*P* = 2.4 × 10^−5^, *D* = 0.25, KS test) and no significant difference between AFR and SEA populations (*P* = 0.21, *D* = 0.13, KS test). We were concerned that these trends in size could be driven by location specific CNVs. We therefore reanalyzed the data including only CNVs (8 deletions and 11 amplifications) that were present in all three continents. For each CNV locus, we took the mean CNV size seen in each continent and compared the distributions of all CNV loci between continents (fig. 3*G*). This showed that CNVs in SAM isolates were significantly longer than those in either SEA (*t* = 4.31, *P* = 1.3 × 10^−4^, *t*-test) or AFR (*t* = 4.23, *P* = 1.6 × 10^−4^, *t*-test) and that CNVs in SEA isolates were longer than in AFR isolates (*t* = 4.02, *P* = 2.9 × 10^−4^, *t*-test).

**Fig. 4.**
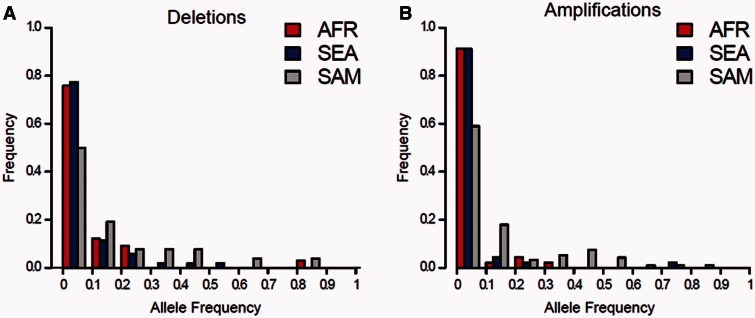
**Allele frequency distribution of (*A*) deletions and (*B*) amplifications.** Allele frequency distributions differ between continents. These distributions fit a neutral model in SAM but not in AFR or SEA (see supplementary fig. S5, Supplementary Material online).

There was a skew toward rare CNVs (both deletions and amplifications) in SEA and AFR populations (fig. 4) with approximately 80% of all variants present at population frequencies of < 10%. However, SAM populations show significantly less skew and show greater consistency with a neutral population structure. We performed coalescent simulations to determine whether the observed CNV allele frequency spectra in each population differ substantially from neutral expectations (supplementary fig. S5, Supplementary Material online). In both SEA (χ*^2^* = 15.2, *P* = 4.94 × 10^−4^, χ*^2^*test) and AFR (χ*^2^* = 8.8, *P* = 0.0121, χ*^2^* test) populations, we can reject neutrality, though SAM does not show a significant deviation from a neutral population structure (χ*^2^* = 2.7, *P* = 0.257, χ*^2^* test). Hence, the increased proportion of high frequency CNVs in SAM, relative to AFR or SEA, contribute to the higher burden of both deletions and amplifications in SAM.

**Fig. 5.**
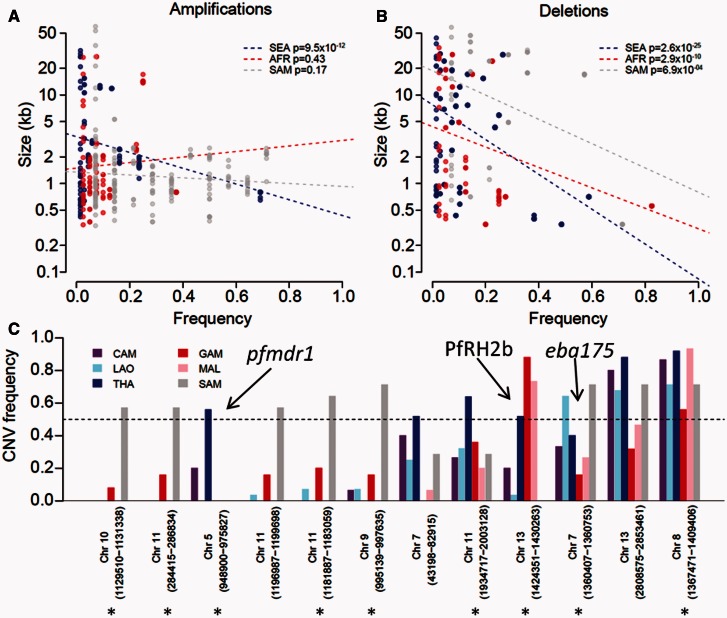
**The relationship between size and frequency of CNVs**. (*A, B*) A negative relationship between size and frequency is seen for deletions but only in SEA for amplifications. (*C*) Twelve CNVs were at > 50% frequency in at least one population. Three of 12 (25%) are known targets of positive selection (*eba175*, *pfmdr1*, PfRH2b), whereas 7/12 (58%) show high *F*_ST/_*G*’_ST_ (marked by asterisk; see figs. 6 and 8).

Under a deleterious model of CNV evolution, we might expect longer CNVs to be more deleterious than short CNVs, so CNV size and frequency should be inversely related. This is clearly observed for deletions in all continents (*P* = 2.6 × 10^−25^ [SEA], *P* = 2.9 × 10^−10^ [AFR], *P* = 6.9 × 10^−4^ [SAM]). However, for amplifications a significant negative relationship was observed for only SEA (*P* = 9.5 × 10^−12^), and no trend was observed in AFR and SAM (fig. 5*A* and *B*).

In total, 22 of 30 tests conducted revealed significant differences in burden, size, or frequency spectrum of CNVs between continents. After Bonferroni correction for multiple testing, this was reduced to 19/30 tests.

### Positive Selection on CNVs

#### Allele Frequency Tests

**Fig. 6.**
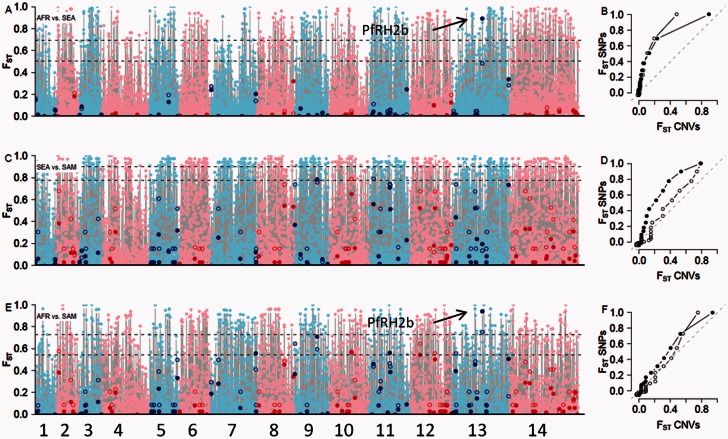
*F*_ST_ **identifies putative targets of natural selection at a continental level**. Genome-wide *F*_ST_ for all biallelic SNPs (pink/light blue dots) and CNVs (red/blue dots) for AFR versus SEA (*A*), SEA versus SAM (*C*), and AFR versus SAM (*E*). The dotted line shows the 90% and 95% thresholds. Open dots are *F*_ST_ calculated from binary classification of CNV, while closed dots utilize CNV size information (*G*’_ST_). The joint distribution of *F*_ST_ for SNPs (*y* axis) and CNVs (*x* axis) is shown by quantile–quantile plots (*B*,*D*,*F*). If the distribution of *F*_ST_ for CNVs and SNPs is equivalent (as could be expected under neutrality), points should lie along the diagonal dash line.

**Fig. 7.**
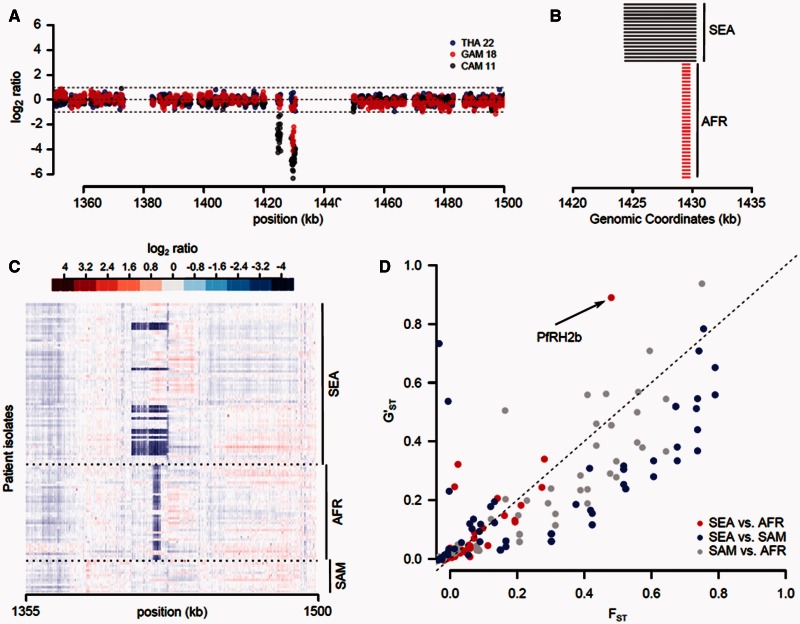
**Deconvolution of the PfRH2b locus results in clearer evidence of selection.** (*A*) Normalized, probe level signal from three representative hybridizations. Boundaries for calling CNVs are shown by dashed lines. THA 22 (blue dots) contains no CNV, while both GAM 18 (AFR) and CAM 11 (SEA) exhibit CNV. The deletion for CAM 11 is larger (5.9 kb vs. 0.5 kb) and includes the neighboring gene, pfRH6. (*B*) Probe signal at this locus for all 122 isolates. Darker blue denotes decreasing probe signal. (*C*) Size variants of the PfRH2b deletion in SEA and AFR, showing difference in size of the deletion present on each continent. No CNV at this locus was seen in SAM. (*D*) Incorporation of CNV size information refines analysis of selection. When CNV size information is incorporated, a net reduction in *F*_ST_ is seen. However, we see an increase in *F*_ST_ at the PfRH2b locus between AFR and SEA.

Given that strong purifying selection appears to act on most CNVs, CNVs present at high frequency within one or more populations sampled are strong candidates for positive selection. We first plotted CNVs by population frequency across the genome (fig. 5*C*). Twelve CNVs show frequencies >50% in at least one population. These included *pfmdr1*, PfRH2b, and histidine-rich proteins II and III. We then searched for CNVs showing divergent allele frequencies between populations by plotting *F*_ST_ across the genome for the three continental comparisons for both SNPs and CNVs genotyped on our array. The distribution of the *F*_ST_ values for CNVs and SNPs can be described by their quantiles (fig. 6*B*, *D*, and *F*, open dots). These show that CNVs are skewed toward lower fixation indices than SNPs between AFR and SEA (AFR: *D* = 0.42, *P* = 3.3 × 10^−13^; SEA: *D* = 0.26, *P* = 4.8 × 10^−7^ KS test). In contrast, comparisons with SAM reveal a smaller, though still statistically significant, skew in the distribution of *F*_ST_ values for both CNVs and SNPs (*D* = 0.20, *P* = 1.1 × 10^−4^ KS test). We used the distribution of *F*_ST_ at SNPs for detection of outlying CNVs. We calculated *F*_ST_ for CNVs using both biallelic classification (CNV vs. WT) or by using size of CNVs to define different alleles (38/135 [28.1%] CNVs had >2 alleles). We used an *F*_ST_-like statistic (*G*’_ST_ [Hedrick 2005]) for multiallelic CNVs (fig. 6, closed dots). This minimizes downward bias in estimates of differentiation and allows comparison between binary and multiallelic CNVs. When we included the size of amplified or deleted regions in *F*_ST_ calculations, we observed a net change in *F*_ST_ statistics at some loci (mean change = 0.044, SD = 0.16, fig. 7*D*).

Using a stringent 95% SNP threshold, we found a single CNV that was significant in one or more continental comparison (PfRH2b, fig. 7). When we relaxed this threshold to 90% (table 1), we identified no additional loci in the AFR-SEA, 3 in the SEA-SAM and 10 in the AFR-SAM comparisons. Three loci were significant in multiple comparisons. As selection acting on nonsynonymous SNPs may drive *F*_ST_ signal, we reanalyzed the data after excluding nonsynonymous variants from the analysis (supplementary fig. S6, Supplementary Material online). This resulted in a minor deviation in the distribution of *F*_ST_ values, though did not alter the inference of selection acting on any CNVs. A deletion encompassing reticulocyte binding-like homologue protein 2 (PfRH2b), a key adhesive molecule involved in erythrocyte invasion, showed significant *F*_ST_ in both the AFR-SEA and AFR-SAM comparisons. Two size variants of this locus are present globally. A small deletion truncates the coding sequence of PfRH2b, whereas a larger variant encompasses the PfRH6 pseudogene upstream of PfRH2b (fig. 7*A–C*). This locus was significant with the multiallelic classification (*G*’_ST_ = 0.89) but not with a biallelic classification (*F*_ST_ = 0.48) in the AFR-SEA comparison, demonstrating the importance of defining CNV alleles, rather than grouping CNV alleles with different origins. The PfRH2b CNV has been extensively studied and evidence suggests that it is under strong selection (Jennings et al. 2007; Lantos et al. 2009; Ahouidi et al. 2010). In addition, two loci, a membrane protein on chr. 9 (PF3D7_0924500) and a conserved gene on chr. 10 (PF3D7_1027000), show significant *F*_ST_ in both SEA-SAM and AFR-SAM comparisons.

**Table 1.** **Summary of CNV Burden in Different Parasite Populations**.

**Country**	**Continent**	***n***	**Number of CNVs**		**CNVs per Parasite**		**No. Genes Affected/Parasite**	
			**Amp**	**Del**	**Amp**	**Del**	**Amp**	**Del**
The Gambia	AFR	25	38	13	3.60	3.80	5.08	7.88
Malawi	AFR	15	8	14	1.27	4.67	1.33	12.33
	AFR	40	46	27	2.73	4.13	3.68	9.55
Thailand	SEA	25	6	18	2.24	5.64	4.64	15.48
Laos	SEA	28	30	24	2.43	4.71	3.17	11.57
Cambodia	SEA	15	12	17	2.60	5.60	4.07	13.67
	SEA	68	48	59	2.40	5.25	3.91	13.47
Venezuela	SAM	12	59	14	13.25	6.17	17.92	28.67
Peru	SAM	2	48	5	29.00	4.50	32.5	13.00
	SAM	14	107	19	14.47	5.53	20.00	26.43

**Fig. 8.**
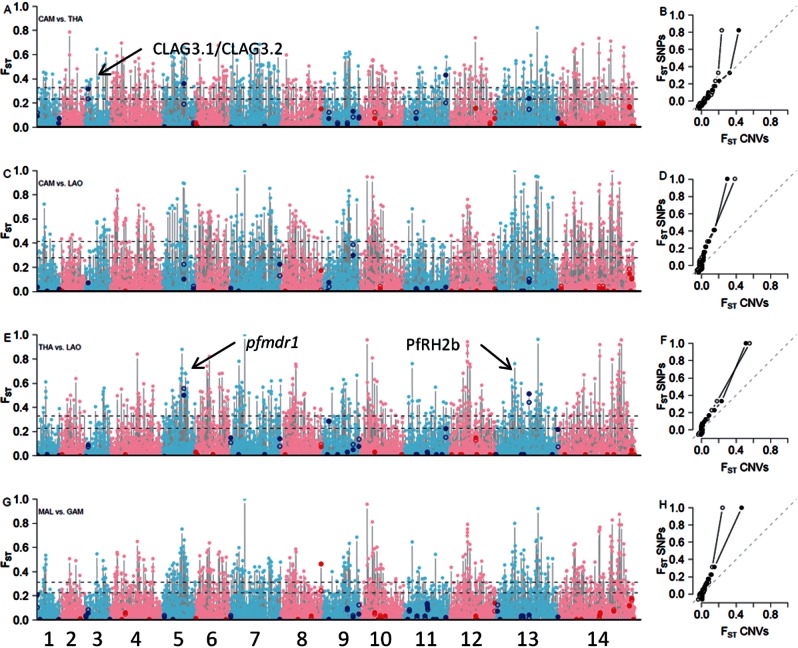
*F*_ST_ **identifies putative targets of natural selection in SE Asia.** Genome-wide *F*_ST_ for all biallelic SNPs (pink/light blue dots) and CNVs (red/blue dots) for CAM versus THA (*A*), CAM versus LAO (*C*), THA versus LAO (*E*), and GAM versus MAL (*G*). The dotted line shows 90% and 95% thresholds. Open dots are *F*_ST_ calculated from binary classification of CNV, while closed dots utilize CNV size information. For the joint distribution of *F*_ST_ for SNPs (*y* axis) and CNVs (*x* axis) is shown by quantile–quantile plots (*B*,*D*,*F*,*H*). If the distribution of *F*_ST_ for CNVs and SNPs is equivalent (as could be expected under neutrality), points should lie along the diagonal dash line.

We conducted a similar *F*_ST_ analysis to compare CNV frequencies from different countries within SEA and AFR (fig. 8; samples sizes in Peru were too small to do this in SAM). The SEA comparison is of particular interest because it has the potential to identify CNVs underlying drug resistance. The three neighboring SEA countries have different histories of antimalarial drug treatment (Anderson, Nair, Sudimack, et al. 2005), and as a consequence, parasite populations differ in the prevalence of drug resistance mutations (Nair et al. 2003, 2007, 2008; Nash et al. 2005; Cheeseman et al. 2012). Both Thailand and Cambodia show wide-spread resistance to multiple antimalarials, whereas Laos populations still retain parasites that are sensitive to all drugs including chloroquine (Mayxay et al. 2007). There were six CNVs in the 90th percentile of SNP *F*_ST_ in comparisons between SEA countries and one CNV in the Malawi–Gambia comparison (table 2). As expected, amplification of the multidrug resistance gene (*pfmdr1*) on chr 5 showed the highest *F*_ST_ in comparisons between Thai and Laos parasite populations. *pfmdr1* is a well-studied locus that is known to be under strong selection (Nair et al. 2007) (fig. 5). PfRH2b also showed highly significant *F*_ST_ in the Thai–Laos comparison, while we found elevated *F*_ST_ in the Cambodian–Thai comparison at a deletion containing *clag3.1/3.2*, genes that determine channel-mediated nutrient uptake by infected red blood cells (Nguitragool et al. 2011) and play a role in blastocidin resistance (Sharma et al. 2013).

**Table:** **Table 2. Putative Positively Selected CNVs**.

**Chr**	**bp coordinates**	**Annotated gene products**	**Significant** *F*_ST_**_/_*G*’_ST_ comparison**^a^
2	140118–141161	Zinc finger protein, putative	AFR-SAM (0.57/0.38)
3	120415–139753	CLAG3.1, pfemp1 psuedogene, unspecified product, CLAG3.2	CAM-THA (0.23/0.32)
5	948900–975827	Zinc finger protein, pfmdr1, mitochondrial processing peptidase alpha, conserved (x2), DnaJ protein	THA-LAO (**0.56**/**0.50**), CAM-THA (0.19/**0.36**)
7	1360407–1360753	Erythrocyte binding antigen 175	AFR-SAM (0.41/0.56)
8	1367471–1409406	Hsp70, PHISTa (x6), hrpII, stevor (x3), rifin (x2)	GAM-MAL (0.24/**0.47**)
9	64226–111135	Exported protein, rifin (x3), stevor, hyp5, FIKK family 9.1-9.6)	AFR-SAM (0.64/0.37)
9	240027–242283	Nucleoporin NUP100/NSP100	THA-LAO (0.28/0.29)
9	995139–997635	Conserved membrane protein	AFR-SAM (0.60/0.71), SEA-SAM (0.76/0.78)
9^b^	1203015–1204856	Msp1	CAM-LAO (0.39/0.30)
10	1129510–1131338	Conserved protein	SEA-SAM (0.79/0.65), AFR-SAM (0.56/0.57)
11	284415–286834	Protein kinase	SEA-SAM (0.79/0.56)
11	1181887–1183059	Structural maintenance of chromosome protein, putative	AFR-SAM (0.47/0.56)
11^b^	1934717–2003128	Ag332	THA-LAO (0.15/0.22), CAM-THA (0.24/**0.43**)
12	501013–502183	DNA helicase MCM8, putative	AFR-SAM (0.64/0.54)
12	1359123–1359544	Conserved protein	AFR-SAM (0.56/0.50)
13	110177–121725	Exported protein (x2), gamete antigen 27/25	AFR-SAM (0.56/0.40)
13	1424351–1430263	Reticulocyte binding protein 2 homologue b	AFR-SEA (0.48/**0.89**), AFR-SAM (**0.75**/**0.94**), THA-LAO (**0.44**/**0.51**), CAM-THA (0.15/0.24)

Note.—These CNVs fell above the 90% threshold for genome-wide SNP comparisons in pairwise comparisons between continents or countries within continents. *F*_ST_ values are presented after size classification.

^a^Comparisons significant at the 95% threshold are underlined.

^b^These loci are included but have intragenic repeat expansions rather than classical CNVs. They are included here for completion.

#### Haplotype Tests

We explored whether strong, recent, positive selection is driving the spread of CNVs using two approaches. First, we measured the integrated haplotype score (iHS) and cross-population extended haplotype homozygosity (XP-EHH) across the genome, treating SNPs and CNVs as equivalent biallelic markers (supplementary figs. S7 and S8, Supplementary Material online). A single CNV (surfin 14.1) in AFR ranked in the top 5% of iHS scores genome wide, while no CNVs ranked in the top 5% of XP-EHH. The chr. 5 amplification containing *pfmdr1* is known to be under strong, recent selection due to its role in mefloquine resistance but was not captured in this genome scan using either statistic. Second, we used a more targeted approach, in which we compared the decay in haplotype homozygosity for the different CNV alleles at each of the putatively selected loci identified from the *G*’_ST_/*F*_ST_ analysis. Our inability to identify selection using long haplotypes surrounding CNVs may have been limited by recurrent mutation of CNVs on different haplotypes. Such a scenario would severely limit the power of haplotype-based tests of selection (Messer and Petrov 2013). We examined EHH surrounding each size delimited allele of a CNV under selection (supplementary fig. S9, Supplementary Material online). We saw little distinction between haplotype decay of individual *pfmdr1* amplicons. This is consistent with previous work, which showed similarly sized amplicons have arisen on distinct haplotypic backgrounds (Nair et al. 2007). At other loci, direct measurement of EHH on size-resolved CNVs did not suggest recent adaptation has acted on any of the CNVs we have identified. We additionally examined if excluding SNP markers which may be subject to selection (nonsynonymous SNPs) altered our ability to identify CNVs with significant iHS scores (supplementary fig. S10, Supplementary Material online). As nonsynonymous SNPs comprise 5,494 of the 10,107 SNPs (53.7%), there was a substantial decrease in marker density. However, exclusion of these SNPs had no impact on the inference of selection acting upon CNVs.

## Discussion

### Validation of a Robust Call Set of CNVs

The *P. falciparum* is extremely AT rich, with highly repetitive telomeric regions containing several multigene families, making it challenging for both sequence alignment (Manske et al. 2012) and analysis of CNVs. We opted to remove problematic regions of the genome from our analysis, to decrease background signal and maximize robustness and repeatability of CNV calling elsewhere in the genome. To this end, we carefully evaluated the extent to which specific genomic features are likely to affect the detection of CNV. By excluding genome regions which exhibit unreliable hybridization signal and applying suitable correction factors (regression against probe AT content and length), we were able to minimize the error rate. However, in doing so, we have limited our analysis to exonic genome regions and excluded gene families, which may possess a high level of CNV. Noncoding regions of eukaryotic genomes have been shown to contain a higher burden of CNV than coding regions (Mills et al. 2011), though the extreme AT content in the malaria parasite (Gardner et al. 2002) genome preclude their analysis here. Similarly, the *pfemp1* (or*var)* gene family and the *rifin* and *stevor* gene families have been suggested to harbor extensive CNV (Freitas-Junior et al. 2000). However, the combination of strong homology among conserved regions of the genes and extensive diversity throughout variable regions (Kyes et al. 2007) makes precise determination of CNV within these complex gene families problematic. Including probes targeting these gene families increases the background probe variation and therefore obscures detection of biologically relevant variation elsewhere in the genome. The resolution of our CNV calling (segments of ≥ 8 probes) puts a practical lower limit on the size of variants we can detect of approximately 500 bp. Previous genome sequencing studies have identified rampant variation in human genomes in the 0–500 bp region (Mills et al. 2011), and further sequencing efforts will be required to elucidate the full spectrum of variation in parasite genomes. However, by limiting the variation we explore in this article to high-confidence polymorphisms, we can make inferences about arguably the most interesting component of the genome coding sequence. These are likely to be both highly visible to both purifying and positive selection and are phenotypically relevant.

### Many CNVs Are Subject to Strong Purifying Selection

Two features of this data set suggest that CNVs tend to be deleterious and subject to strong purifying selection. First, the distributions of CNVs are strongly skewed toward low frequency variants, particularly in AFR and SEA populations (fig. 4, supplementary fig. S5, Supplementary Material online). Second, for deletions, there is a strong negative correlation between deletion size and population frequency (fig. 5*A* and *B*). This pattern is most likely observed because loss of several genes is more likely to have deleterious impact than loss of a single gene. That this correlation is not seen for amplifications suggesting that selection against this class of CNV is less restrictive.

There are several reasons why CNVs may be under particularly strong purifying selection in malaria parasites relative to diploid organisms. First, deletions in essential genes will result in death of haploids, but deleterious deletions can be maintained in heterozygous state in diploids. Similarly, amplifications are expected have stronger dosage effects in haploids than diploids because gene duplication results in 2-fold change in haploids (1→2 copies), while amplification of a single gene copy results in 1.5-fold change (2→3 copies) in diploids. There is strong empirical evidence that gene dosage results in dramatic genome wide change in transcription patterns from an eQTL study of *P. falciparum* (Gonzales et al. 2008). Forty-eight percent of expression quantitative trait loci (eQTLs) identified mapped to the chr 5 CNV containing *pfmdr1*, suggesting that this CNV has risen to high frequency in SE Asian countries due to extremely strong drug selection despite extensive perturbation of the parasite transcriptome.

### Greater Burden of CNVs in South America: Impact of Parasite Demography

A striking feature of these data is that SAM parasites carry a larger burden of CNVs than parasites from SEA or AFR. This contrasts with diversity measured using SNPs and microsatellites, which universally demonstrate reduced variation in SAM parasite populations (Anderson et al. 2000). Each of the continental populations used in our dataset differs in key epidemiological parameters. The effective population size, recombination rate, outcrossing rate, and mean clonality of infections are all highest in AFR and lowest in SAM (Conway et al. 1999; Anderson et al. 2000) and show an inverse relationship to CNV burden (fig. 2). We see a similar scaling of CNV size by continental origin (fig. 3*E–G*), a result we demonstrate is unlikely due to any specific bias of CNV frequency or CNV burden. Coupled with our analysis of the relationship between CNV size and CNV frequency (supplementary fig. S5, Supplementary Material online), this provides support for a model where CNVs are generally under strong purifying selection. As the strength of purifying selection scales with *N_e_*, settings where *N_e_* is reduced (such as SAM) have a reduced ability to purge deleterious CNVs from the population. While in SEA and AFR, we see a skew toward more rare variants than expected under neutrality (supplementary fig. S5, Supplementary Material online), no clear deviation is seen for SAM populations. This model is also supported by forward simulations of a locus under weak purifying selection in populations with different *N_e_* (supplementary fig. S11, Supplementary Material online). The trajectory of a weakly deleterious mutation in a large population is largely deterministic and rapidly driven to lower frequencies, while smaller populations maintain deleterious variants for longer periods, and these may drift to higher frequency. These simulation results demonstrate that the differences in *N_e_* observed in malaria parasite populations are sufficient to explain the differences in CNV burden, size, and distribution between populations.

The patterns observed in malaria parasites are consistent with those observed in studies of the impact of demography on the distribution of deleterious SNP alleles in human populations. A high incidence of recessive diseases is found in populations that have passed through recent bottlenecks such as Finns and Ashkenazi Jews (Savukoski et al. 1998; Aaltonen and Bjorses 1999; Aminoff et al. 1999) compared to other European populations. Similarly, on a genome-wide scale, Finnish populations show fewer SNPs overall than non-Finnish European populations, but they show higher frequencies of loss-of-function variants and complete gene knockouts than non-Finnish Europeans (Lim et al. 2014). Comparison of African American and European American populations is also informative. A large exome sequencing study revealed that European Americans have a large excess of deleterious variants in essential and Mendelian disease genes compared to African Americans, consistent with weaker purifying selection resulting from smaller *N_e_* in European American ancestral and/or founder populations (Fu et al. 2013).

We suggest that differences in *N_e_* provide a likely explanation for the observed patterns, but this is not the only possible explanation. Several key population parameters covary with transmission intensity and *N_e_* in *Plasmodium*. For example, in low transmission settings, parasite gametes of the same genotype typically fuse and recombine within the mosquito, resulting in high levels of inbreeding, while outbreeding is more common in high transmission regions (Anderson et al. 2000). One alternative explanation for the high burden of large CNVs in SAM is that these cannot be efficiently purged from populations by recombination. Similarly, selection regimes experienced by parasites differ in high and low transmission settings and differences in CNV burden could be driven by adaptation. Parasites are under strong drug selection in low transmission regions because patients are usually symptomatic and seek treatment, while in Africa, drug selection is weak because most infected people are asymptomatic and do not seek treatment. We think this explanation is unlikely, because 1) most CNVs are rare, 2) we found rather few high frequency CNVs consistent with positive selection, 3) SEA and SAM populations are both exposed to strong drug selection but show strong differences in CNV burden, and 4) removal of known CNVs associated with drug resistance did not alter the genome-wide patterns observed.

### Positive Selection Acting upon CNVs

If purifying selection acts strongly against most CNVs in malaria parasites, this suggests that CNVs which have risen to high frequency (Aminetzach et al. 2005), or those that show divergent allele frequencies in different countries, may be subject to positive selection. Our study reveals 12 CNVs that have risen to > 50% in any one parasite population. Furthermore, we found just 11 CNVs showing significant intercontinental *F*_ST_ and 7 showing significant intracontinental *F*_ST_. Several of the CNV loci show independent evidence for positive selection in previous studies and provide useful positive controls. These include a well-studied amplification on chr. 5 containing *pfmdr1* (chr. 5), which underlies resistance to several antimalarial drugs (Nair et al. 2007), and the reticulocyte binding protein 2 homolog b (PfRH2b), which is involved in parasite invasion of erythrocytes (Rayner et al. 2000; Duraisingh et al. 2003). Another gene amplification on chr. 12 containing the GTP cyclohydrolase I gene is also known to be under strong positive selection and plays a role in evolution of antifolate resistance (Nair et al. 2008; Heinberg et al. 2013; Kumpornsin et al. 2014). This locus was not detected in our survey, but the reason is technical. The 3D7 parasite which we used as a reference for all our hybridizations has 4–5 copies of this amplification, so we were unable to score this CNV in our population samples.

**Fig. 9.**
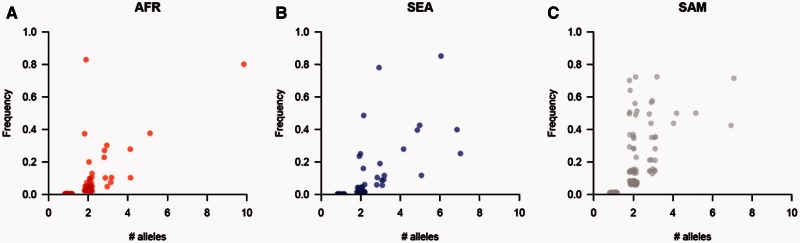
**The relationship between CNV frequency and the number of alleles at a locus.** We see a positive correlation between the frequency of a CNV and the number of size polymorphisms observed in that CNV for African (*A*), SE Asian (*B*), and South American (*C*) populations.

The rapid spread of beneficial CNV alleles is expected to result in detectable genomic signatures in the form of long-flanking haplotypes. Our analyses failed to detect such signatures. First, when we examined genome-wide plots of haplotype tests (iHS, XP-EHH) just one CNV (containing surfin 14.1) fell in the top 5% of values for iHS and none for XP-EHH. Second, we found no significant differences in haplotype length surrounding CNV and wild-type alleles. We suggest two reasons why haplotype tests may perform poorly for detecting selection around CNVs. First, long-haplotype tests (such as the iHS and XP-EHH tests used here) are based on the classical “hard sweep” model of Maynard–Smith (Smith and Haigh 1974; Sabeti et al. 2002). A key assumption is that mutation is rare, so beneficial mutations have a single origin and a single long haplotype will hitchhike with the beneficial mutation. This is a weak assumption when mutation rate is high, as is expected for CNVs (Lipinski et al. 2011), and when populations are large, as for microbial pathogens such as malaria (Pennings and Hermisson 2006; Pritchard et al. 2010). In this case, multiple independent origins of beneficial CNVs are expected, so long-haplotype tests will be underpowered. There is strong empirical evidence that beneficial gene amplifications have multiple independent origins in malaria parasites (Nair et al. 2007, 2008). Five to 15 independent origins of *pfmdr1* amplification are recorded in a single Thai location and have created a “soft sweep” surrounding this gene (Pennings and Hermisson 2006; Nair et al. 2007), while three independent origins of GTP-cyclohydrolase I amplifications were observed in the same population (Nair et al. 2008). Interestingly, of the CNVs reaching over 50% frequency in any sampled population, 8/12 (66.7%) had amplicons (or deletions) that differed in size and gene content, consistent with independent origins. For example, we classified *pfmdr1* amplicons by their target sequence and detected 6 amplicons (10–24 kb in length) each found in 1–9 parasites (fig. 9, supplementary fig. S9, Supplementary Material online).

A second characteristic of CNVs may also complicate the use of long haplotype tests. Unlike point mutations, gene amplifications are easily reversible. As a consequence, beneficial duplications may spread within populations but then back-mutate to single copy status. As consequence, long haplotype may be associated with both duplications and single copy genes. We see evidence that this occurs for both GTP-cyclohydrolase I and *pfmdr1* (Nair et al. 2007; Anderson et al. 2009). These results suggest caution when using long haplotype tests for implicating CNV as the target of positive selection.

## Materials and Methods

### Collection of Malaria Parasites and Preliminary Genotyping

We obtained parasite-infected blood from *P. falciparum-*infected patients from three countries in Asia (Thailand, Laos, and Cambodia), from two countries in Africa (Malawi and the Gambia), and from two countries in South America (Venezuela and Peru) (supplementary table S1, Supplementary Material online). In each location, we collected 6–10 ml blood from patients with >0.5% parasite density to provide sufficient parasite DNA for genetic characterization, and patients coinfected with other *Plasmodium* species were excluded. After removal of the buffy coat, DNA was extracted using the phenol-chloroform method and parasite DNA concentrations estimated by real-time polymerase chain reaction of the *ama-1* parasite gene. All samples fell within acceptable limits of human DNA contamination empirically determined in Tan et al. (2011).

Parasite isolates were prescreened for multiple infections and identical clones using previously collected genotype data. Thailand, Laos, and Malawi samples were genotyped using seven polymorphic microsatellite markers (*ARA2* (chr 11), *POLYα* (chr 4), *TA1* (chr 6), *C2M1* (chr 2), *C3M54* (chr 3), *TA60* (chr 13), and *C4M30* (chr 4), which were amplified and scored on a capillary sequencer (Anderson, Nair, Qin, et al. 2005). Cambodian samples were genotyped with 18 microsatellite loci (Anderson et al. 2010). South American samples were genotyped for 96 SNPs using the Veracode platform (Illumina) (Phyo et al. 2012), whereas Gambian samples were genotyped with two highly polymorphic antigen loci (Merozoite surface antigens 1 and 2) (Snounou et al. 1999). These preliminary genotyping data enabled us to 1) to identify and exclude infections containing more than one parasite genotype. Such multiple genotype infections (MGI) are particularly common in African and Asian locations and preclude accurate genotyping of parasite haplotypes. 2) To identify genetically indistinguishable infections in different patients. These parasite clones occur frequently in populations with low transmission where selfing between male and female parasites from the same infections occurs (Anderson et al. 2000, 2010; Echeverry et al. 2013). To minimize relatedness in our sample, we retained one parasite genotype from each clonal group.

### Ethics Statements

We obtained written informed consent from all participants in this study. In the case of minors, the parents or guardians gave consent prior to enrollment. Consent procedures, study protocols, and collection of malaria-infected blood samples in each country was approved by the appropriate national and international ethics review boards as follows:

The Gambia: The Gambia Government and MRC Joint Ethics Committee, and the Ethics Committee of the London School of Hygiene and Tropical Medicine.
Malawi: College of Medicine Research and Ethics Committee, University of Malawi.
Thailand: Ethics Review board of the Faculty of Tropical Medicine, Mahidol University.
Cambodia: Ministry of Health in Cambodia, the Oxford Tropical Medicine Ethical Committee, the World Health Organization (WHO) Research Ethics Review Committee, and the Technical Review Group of the WHO Western Pacific Regional Office.
Laos: The National Ethics Committee for Health Research, Ministry of Health, Laos.
Peru: The Institutional Review Boards at New York University, the Peruvian Ministry of Health Institutes of National Health, and the University of Alabama at Birmingham.
Venezuela: The committee on bioethics and biosafety of the Institute for Research in Biomedicine and Applied Sciences (IIBCA), Universidad de Oriente.

### Comparative Genomic Hybridization

These isolates were then hybridized to a custom Nimblegen SNP-CNV genotyping array (Tan et al. 2011) following standard Nimblegen CGH protocols. The array contains 364,192 probes which interrogate 45,524 SNP loci, as well as 50–75 bp cGH probes tiled through the genome. Use of a microarray, rather than Illumina sequencing, allowed us to examine parasite infections with substantial amounts (>95%) human DNA contamination. To accommodate the extreme nucleotide bias in the *P. falciparum* genome (Gardner et al. 2002), 65% AT cy3 and cy5-labeled random nonamers were used to fluorescently label target DNA. Genotype calls were performed as in Tan et al. (2011), and the data were cleaned to remove loci with >20% missing data and a minor allele frequency of < 5%. The accuracy of SNP typing for this platform have been previously tested (Tan et al. 2011; Cheeseman et al. 2012; Manske et al. 2012). CNV calling was performed by segmenting the genome using CGHseg using default settings and classifying gains and losses as regions which had >8 probes and had a log 2 ratio (cy3/cy5) > 2.5 (for amplifications) or 3 (for deletions) times the standard deviation of the underlying probe level data. Prior to analysis probes on the array were remapped to PlasmoDB version 9.2 of the 3D7 reference strain (http://plasmodb.org/common/downloads/release-9.2/Pfalciparum3D7/fasta/data/, last accessed December 11, 2015) using BLAST v2.2.27 where best perfect hits were retained for each probe.

### CNV Calling from Next-Generation Sequencing

Genome sequences of four Thai isolates, that were genotyped on the microarray, were generated as part of a recent, large-scale resequencing effort (Manske et al. 2012). We aligned short reads from each of these to PlasmoDB v9.2 of the 3D7 genome using bwa (v0.6.1) and generated sorted indexed .BAM files using samtools (v0.1.18), and these were used as an input to CNV calling programs. Each CNV calling algorithm was run with default parameters except for the following changes: FREEC v6.0 window size = 200 bp, quadratic polynomial for GC regression, ploidy = 1, min expected GC = 0.05, max expected GC 0.5, CNVnator v0.2.7 bin size = 200. Parameters for BreakDancer v1.1.2 were calculated using the bam2cfg.pl script included in the BreakDancer distribution). We excluded CNVs which fell outside the genome regions called by the array using Bedtools v2.17.0 and additionally filtered out CNVs < 500 bp long and amplifications with a copy number of < 4 (FREEC), a median ratio of > 1.75 (amplifications) or < 0.5 (deletions) and a q0 of > 0.5 (CNVnator) and structural variations which were not classified as either deletions or insertions (BreakDancer).

### Statistical and Population Genetic Analysis

All statistical analysis were performed in R v3.0.2 except for *F*_ST_ analysis which was performed in R2.6.2 using the hierfstat package (GOUDET 2005). *G*’_ST_ was calculated using the SMOGD v.1.2.5 software (Crawford 2010). Significant *F*_ST_ values for CNVs were determined by taking the *F*_ST_ distribution of SNPs as a null distribution and scoring CNVs which fell outside of the 95^th^ percentile as significant. Kolmogov–Smirnov and *t*-tests were two tailed and performed using the stats package. Survival analysis of CNVs was performed using the Survival package.

Neutral allele frequency spectra were generated using msms v3.2. following Cridland and Thornton (2010) using the options -ms Nchr Nreps –t *θ_w_* –s Segsites -oAFS, where Nchr is the number of chromosomes simulated (equal to the sample size: 40,68,14), Nreps is the number of simulations to perform (1,000), *θ_w_* is Watterson’s theta and Segsites is the number of segregating sites in the population. The allele frequency spectra were output for comparison to actual data.

### Data Access

Array data have been submitted to the NCBI Gene Expression Omnibus (GEO; http://www.ncbi.nlm.nih.gov/geo/, last accessed December 11, 2015) under accession numbers GSE75137.

## Supplementary Material

Supplementary figures S1–S11, data set S1, and tables S1 and S2 are available at *Molecular Biology and Evolution* online (http://www.mbe.oxfordjournals.org/).

Supplementary Data
