# Supplementary material for: Population Structure Shapes Copy Number Variation in Malaria Parasites
Source: Mol Biol Evol. 2015 Nov 26;33(3):603–20. doi: 10.1093/molbev/msv282 (PMC4760083; doi:10.1093/molbev/msv282)
Supplement: Supplementary Data [file supp_33_3_603__index.html]

Population Structure Shapes Copy Number Variation in Malaria Parasites — Population Structure Shapes Copy Number Variation in Malaria Parasites — Supplementary Data 

# Population Structure Shapes Copy Number Variation in Malaria Parasites

## Supplementary Data

files

- Supplementary Data - zip file
