# Supplementary material for: Population Structure Shapes Copy Number Variation in Malaria Parasites
Source: Mol Biol Evol. 2015 Nov 26;33(3):603–20. doi: 10.1093/molbev/msv282 (PMC4760083; doi:10.1093/molbev/msv282)
Supplement: Supplementary Data [file supp_msv282_suppl_data.zip › Supplemental Figure Legends_TA.docx]

**Supplemental Figure Legends**

**Supplementary Figure 1. Principal Component Analysis (PCA).** PCA of 10,107 polymorphic SNPs demonstrates the extent of global parasite population structure. Parasites cluster by their country and continent of origin.

**Supplementary Figure 2. Quality control filtering of hybridizations.** Parasite isolates showing high level of variance in probe hybridization (s.d. < 0.42, see dotted line threshold in SD plot) were excluded from the analysis. This threshold was selected after empirical evaluation of raw hybridization plots.

**Supplementary Figure 3. CNVs called by the CNV/SNP array and by genome sequencing.** The Venn diagram shows the CNVs called arrays, compared with those called by three different deep sequence based algorithms (FREEC, Breakdancer and CNVnator) for 4 parasite isolates for which deep sequence and hybridization data were available. Ninety-six percent (114/119) of the CNVs detected by CGH overlap with CNVs detected by one or more method in the sequence data.

**Supplementary Figure 4. Genome-wide map of CNVs detected in this study.** Each row denotes a single isolate colored by population (red=The Gambia, pink=Malawi, grey=South America, purple=Cambodia, light blue=Laos PDR, blue=Thailand).See supplementary table 2 and supplementary dataset 1 for more details. The inset shows the relationship between chromosome size and number of CNVs detected.

**Supplementary Figure 5. The allele frequency spectra of CNVs rejects neutrality in AFR and SEA.** We compared a neutral frequency spectra generated by coalescent simulations to our observed data. We were able to reject neutrality in both AFR and SEA populations, suggesting the observed allele frequency spectrum for CNVs in these locations is driven by selective and demographic influences.

**Supplementary Figure 6.** Empirical cumulative distribution functions of F_ST_ for each population comparison. For each plot the distribution from non-coding, synonymous and non-synonymous SNPs are shown separately. There was no significant difference in the distribution of FST from synonymous and non-coding SNP to the combined distribution.

**Supplementary Figure 7. Genome-wide iHS for each population.** Values from SNPs are shown in red and black (for alternating chromosomes) and CNVs in purple. Dashed lines show empirical 5% thresholds for the SNP dataset. Each continent (AFR (A), SEA (B) and SAM (C)) were independently measured. Just one CNV falls in the 95th percentile.

**Supplementary Figure 8. Genome-wide XP-EHH between AFR and SEA populations.** Values from SNPs are shown in red and black (for alternating chromosomes) and CNVs in purple. Dashed lines show empirical 95th percentiles for the SNP dataset. No CNVs fall above or below these thresholds.

**Supplementary Figure 9. CNV size deconvolution, haplotype structure and size specific EHH.** Each of the 6 panels shows a common CNV and the haplotype structure of flanking SNPs. Five of 6 CNVs shown are multiallelic – the size and span of each CNV allele are illustrated, with each different allele color-coded (top left in each panel). SNP haplotypes around CNVs (right in each panel) are shown with each row representing a sample, and color-coding showing the position of each different CNV allele. The EHH plots show the decay in EHH surrounding the different size allele. The solid line shows EHH decay for wild-type alleles (no CNV) while colored dotted lines show EHH decay surrounding different CNV alleles. We did not observesignificantly higher levels of LD around CNVs when compared with wild-type alleles (see also supplementary figure 6).

**Supplementary Figure 10.** iHS scores for CNVs using either all flanking markers, or only non-coding/synonymous markers. No CNVs were above the threshold for classification as under selection following exclusion of non-synonymous markers.

**Supplementary Figure 11. Forward simulation of weak purifying selection in populations with different *N_e_*.** We simulated the trajectory of an allele under weak purifying selection (s≈0.0005) over 1000 generations in 3 distinct populations with Ne=1000000,125000 and 40000. These correspond to the maximal range of *N_e_* observed in contemporary parasite populations (Anderson et al, MBE 2000). In each case the initial allele frequency was set at 5%. After 1000 generations the variance in allele frequencies between populations was 2.4x10^-5^, 3.0x10^-4^ and 5.1x10^-4^.

**Supplementary Table 1. Parasite Sampling.**

**Supplementary Table 2. CNV loci detected in this study.**

**Supplementary Dataset 1. SNPs and CNVs detected in this study.**
