# Supplementary material for: Population Structure Shapes Copy Number Variation in Malaria Parasites
Source: Mol Biol Evol. 2015 Nov 26;33(3):603–20. doi: 10.1093/molbev/msv282 (PMC4760083; doi:10.1093/molbev/msv282)
Supplement: Supplementary Data [file supp_msv282_suppl_data.zip › Supplementary_figures_revised.pptx]

## Slide 1
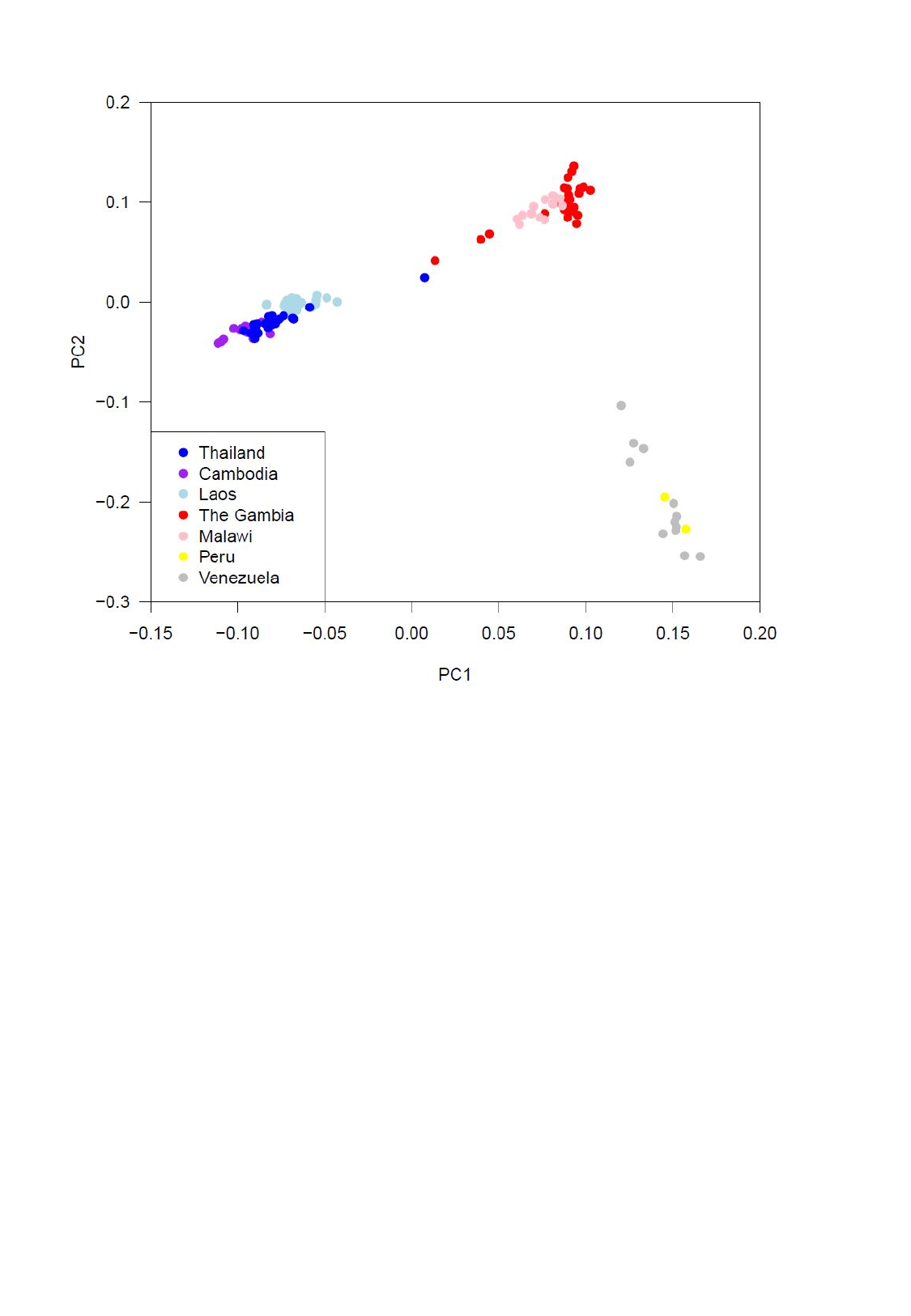

## Slide 2
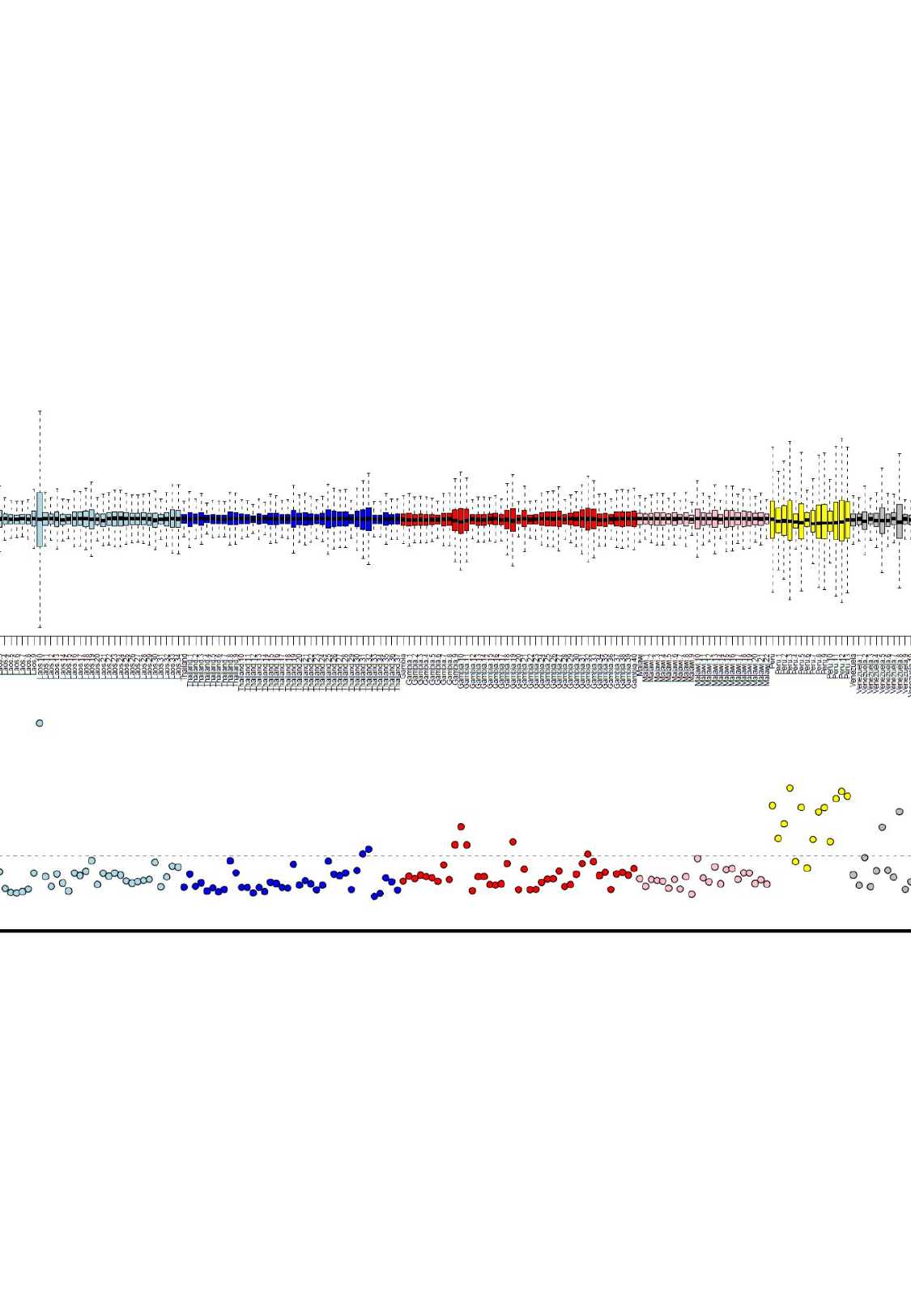

## Slide 3
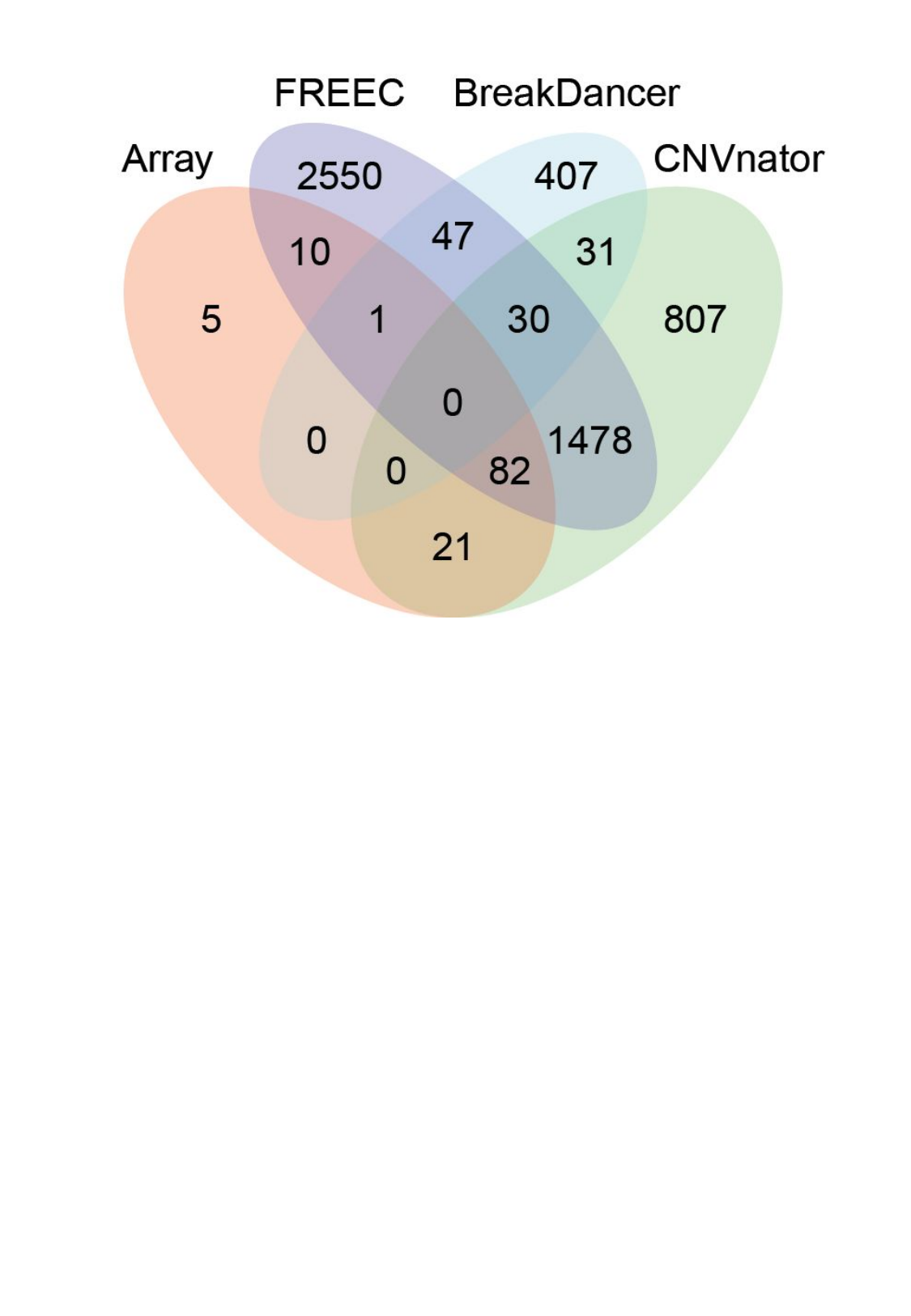

## Slide 4
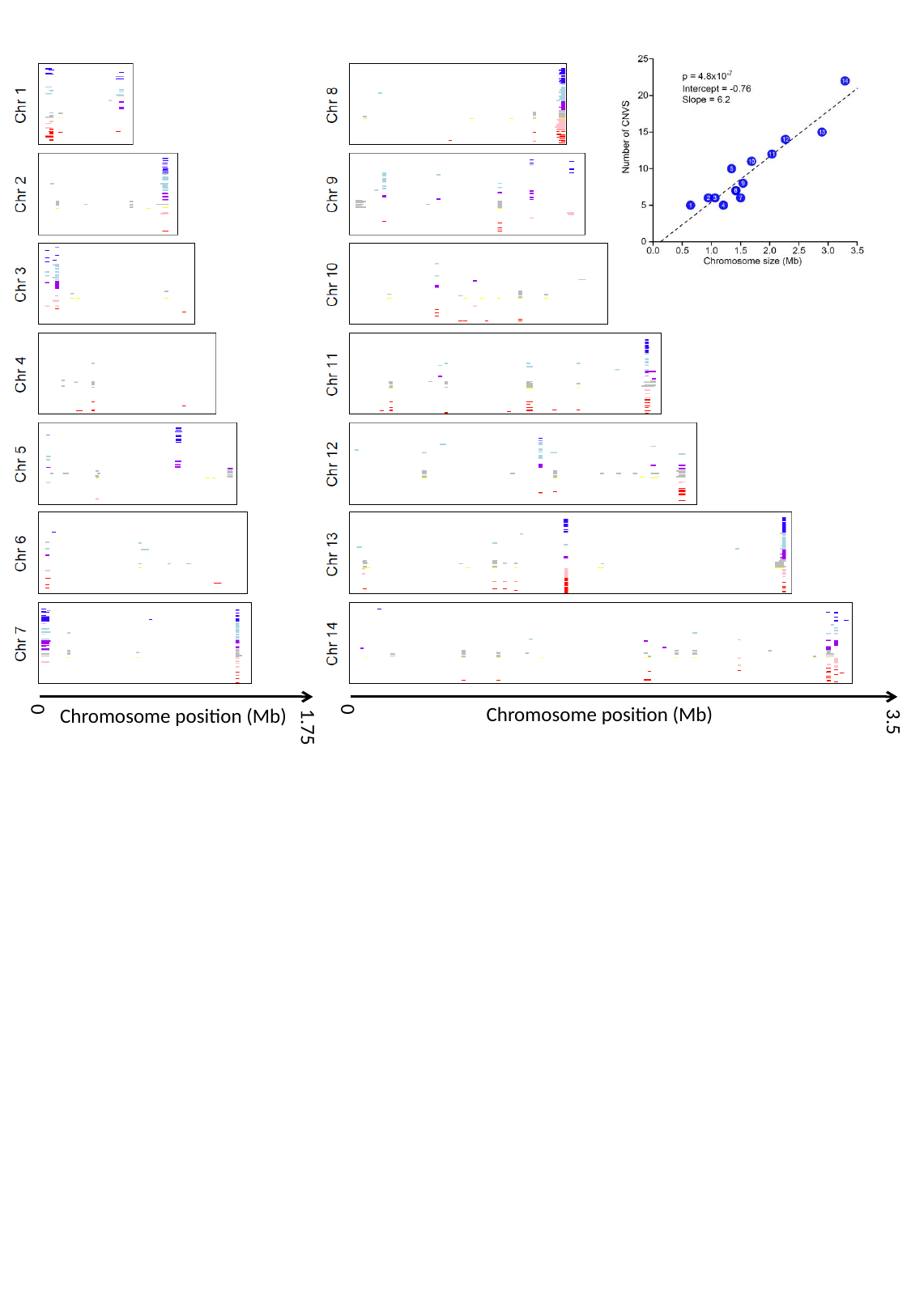

0
0
Chromosome position (Mb)
Chromosome position (Mb)
3.5
1.75

## Slide 5
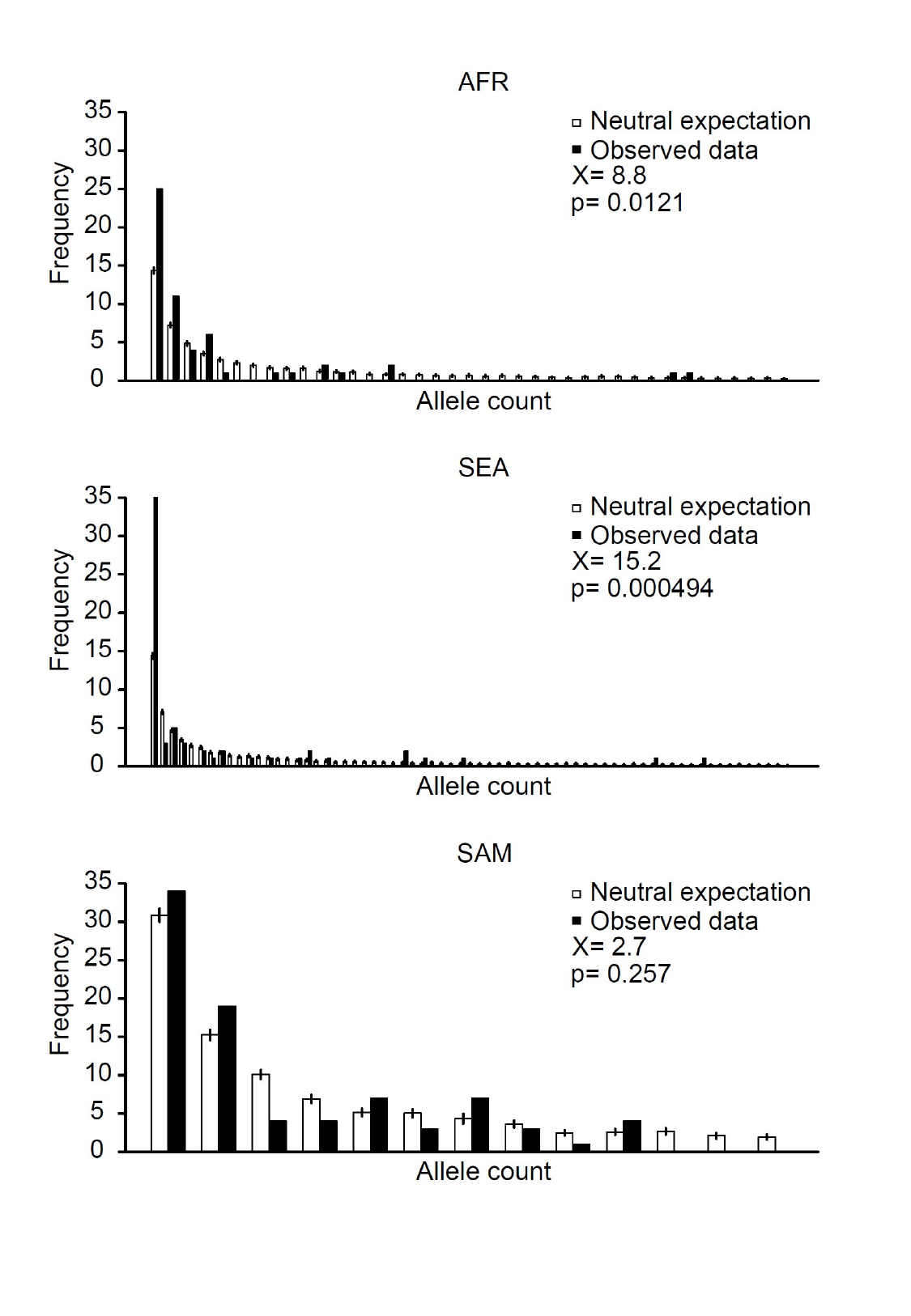

## Slide 6
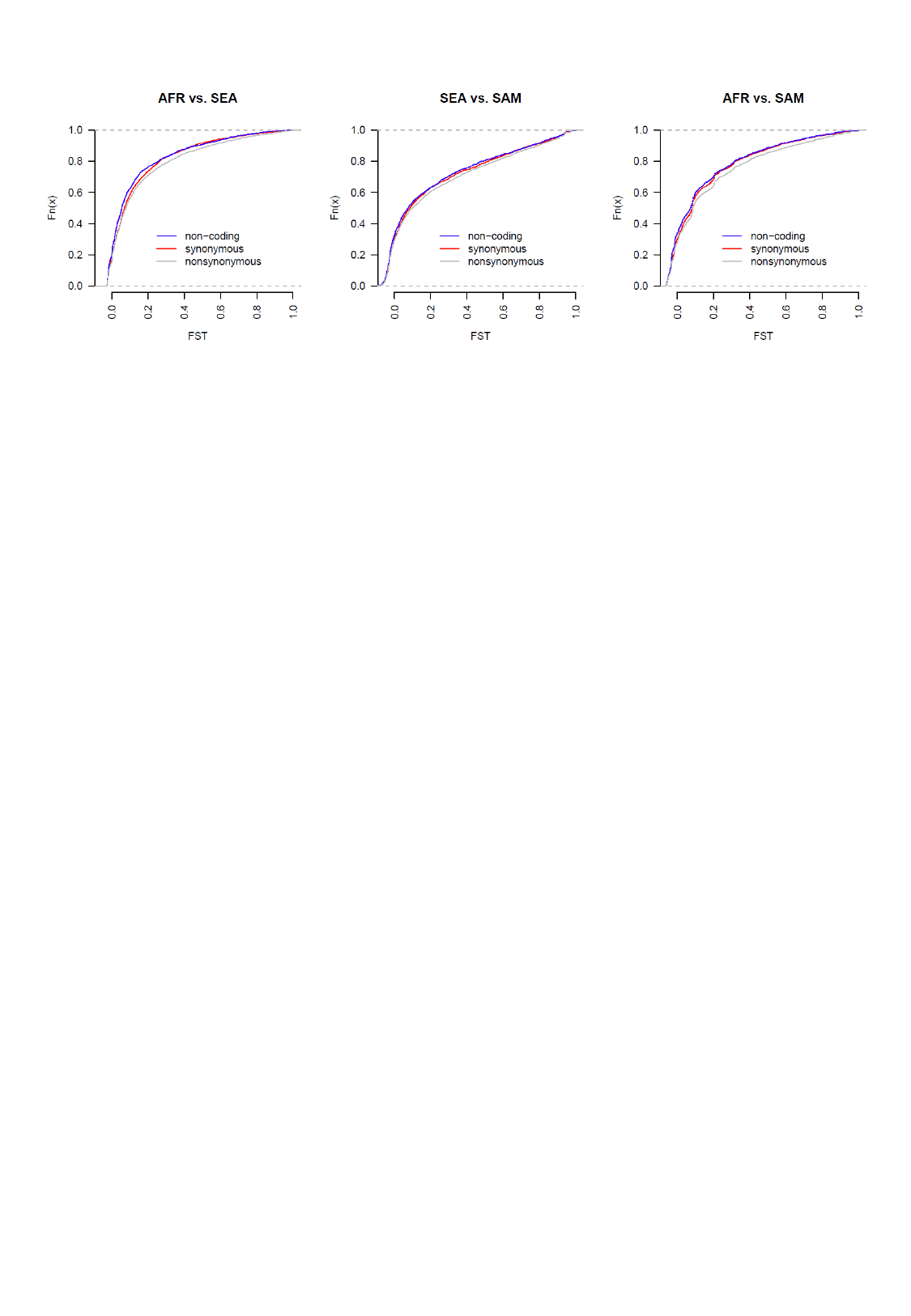

## Slide 7
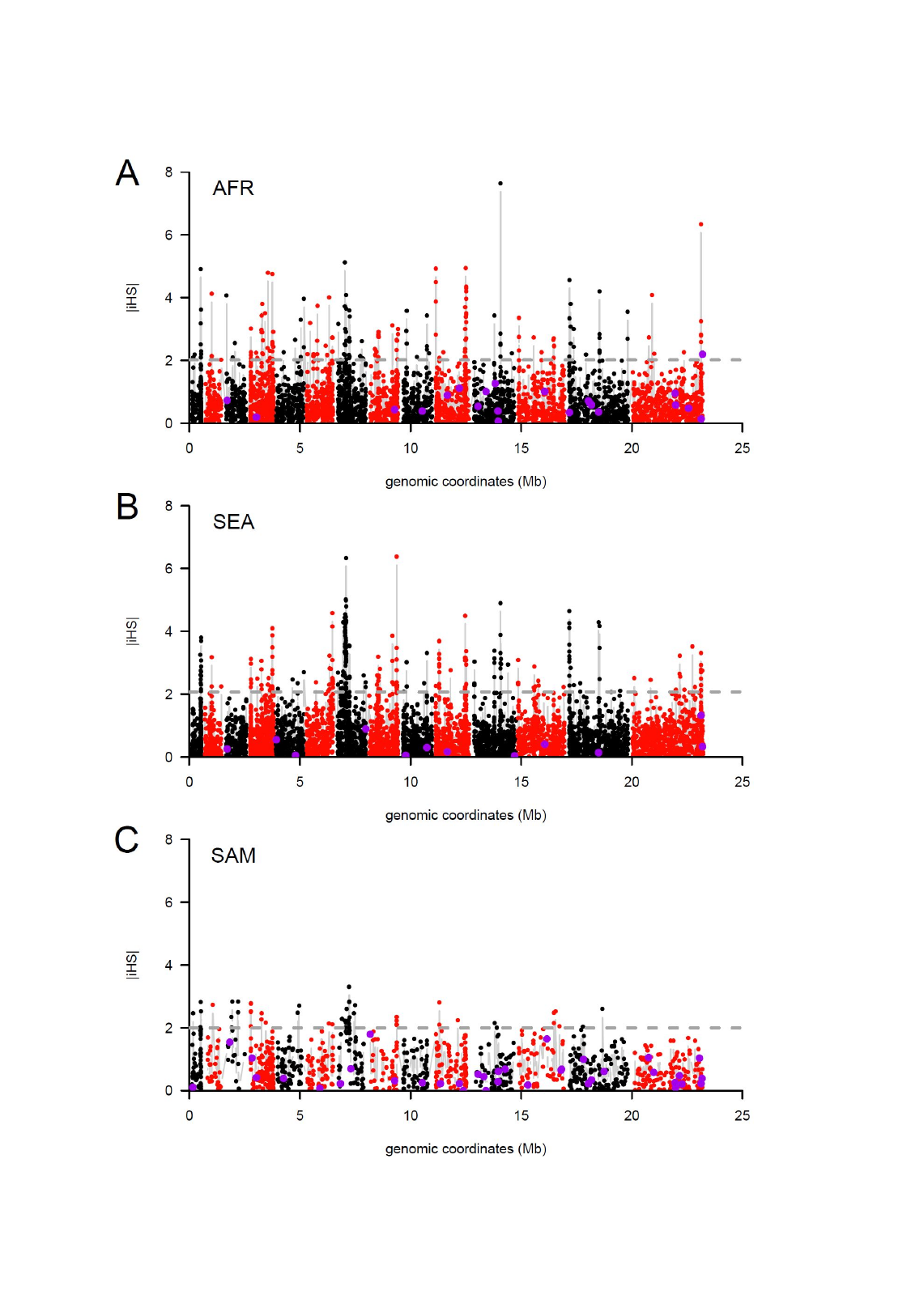

## Slide 8
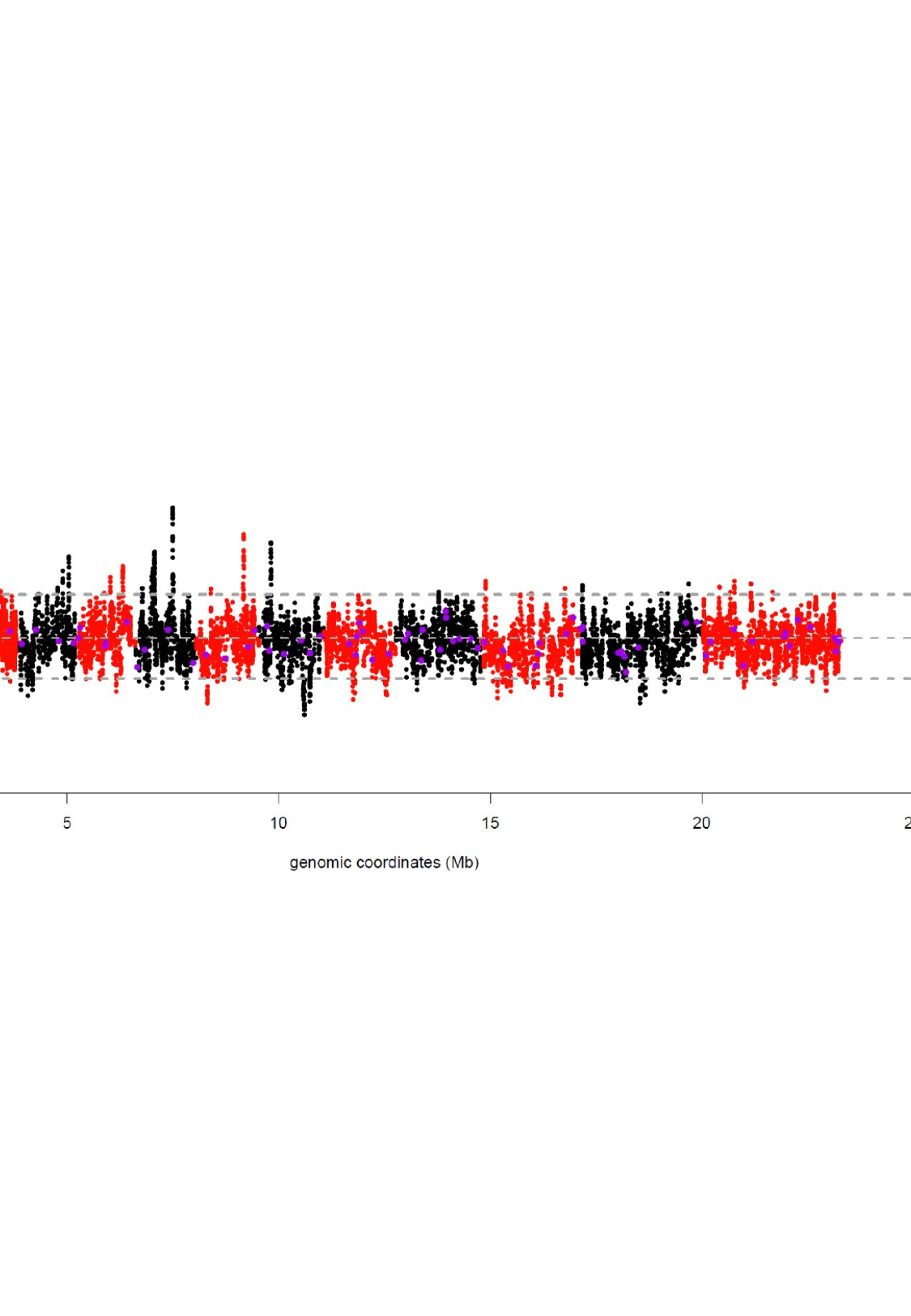

## Slide 9
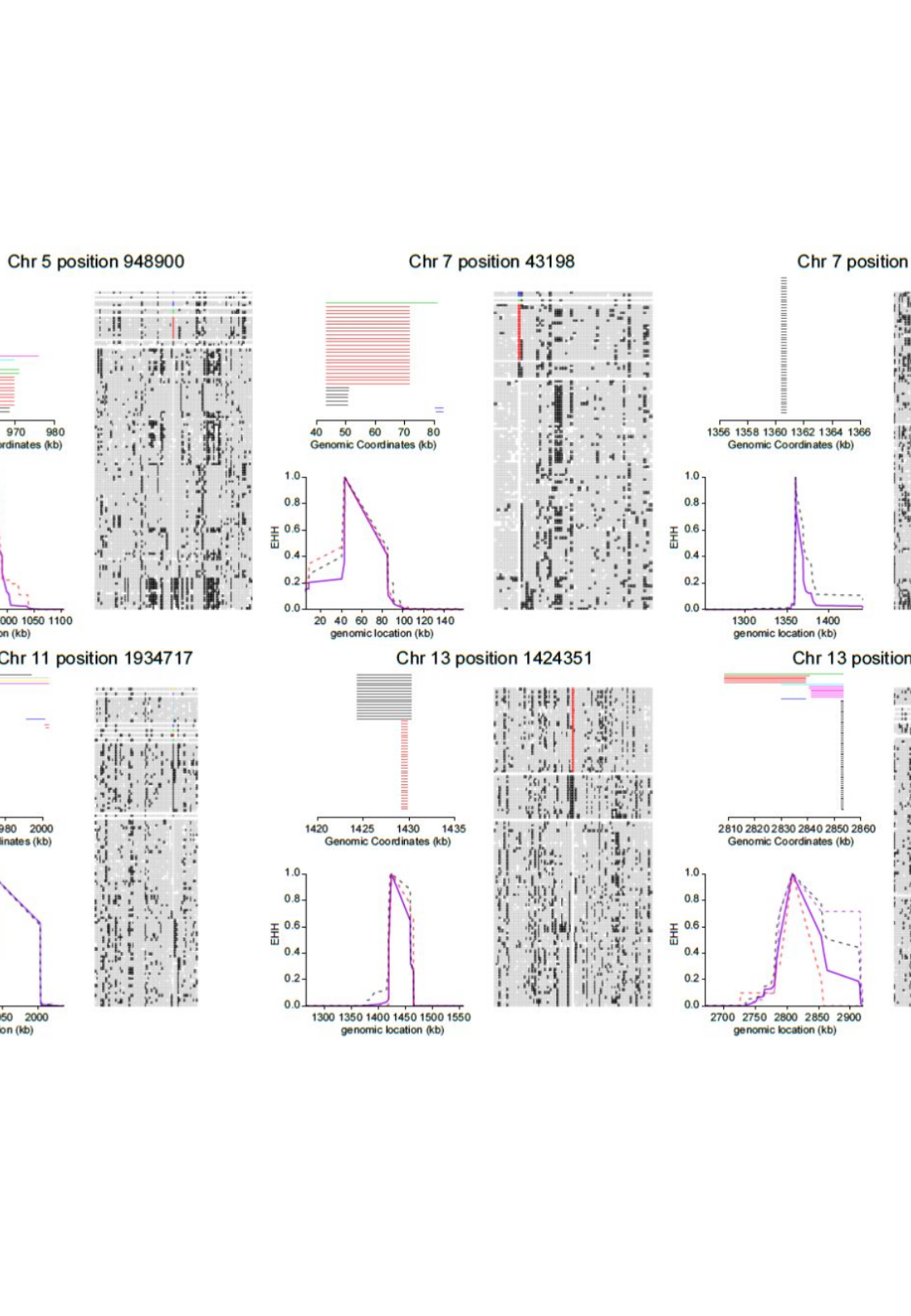

## Slide 10
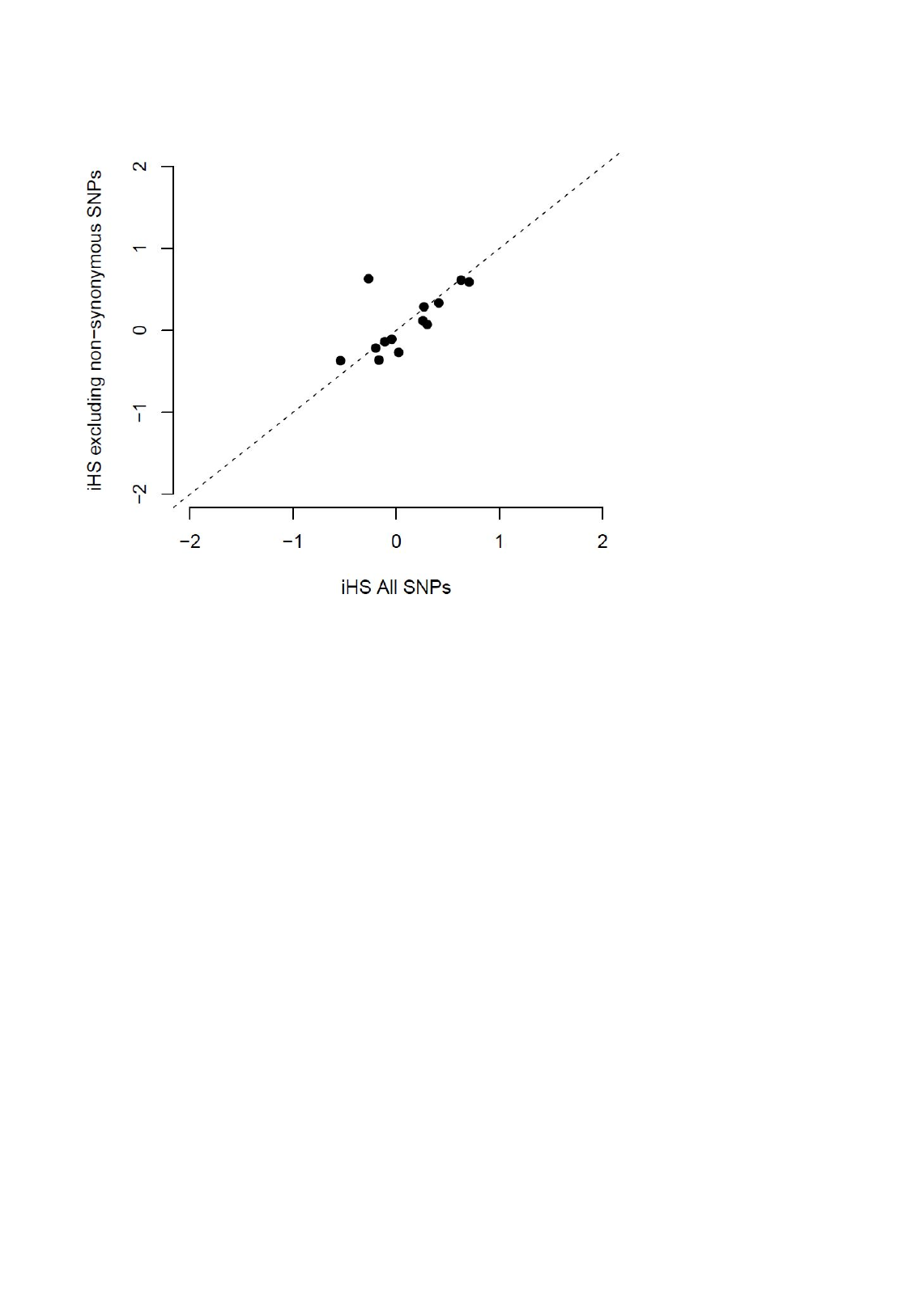

## Slide 11
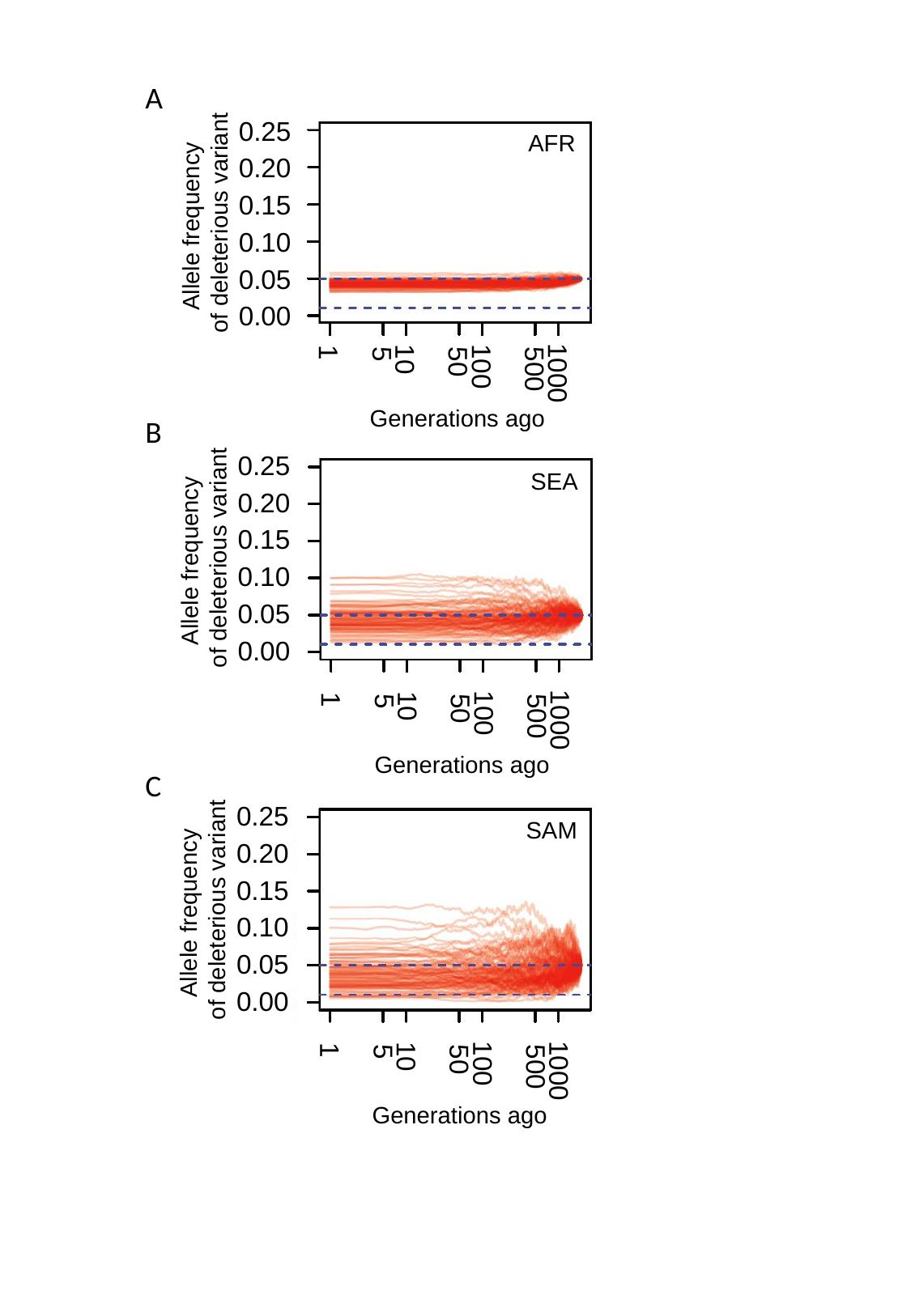

A
0.25
AFR
0.20
0.15
0.10
0.05
0.00
1
5
10
50
100
500
1000
Allele frequency
of deleterious variant
Generations ago
0.25
SEA
0.20
0.15
0.10
0.05
0.00
1
5
10
50
100
500
1000
Allele frequency
of deleterious variant
Generations ago
0.25
SAM
0.20
0.15
0.10
0.05
0.00
1
5
10
50
100
500
1000
Allele frequency
of deleterious variant
Generations ago
B
C
