# Supplementary material for: Population Structure Shapes Copy Number Variation in Malaria Parasites
Source: Mol Biol Evol. 2015 Nov 26;33(3):603–20. doi: 10.1093/molbev/msv282 (PMC4760083; doi:10.1093/molbev/msv282)
Supplement: Supplementary Data [file supp_msv282_suppl_data.zip › Supplementary Table 1.docx]

| **Supplementary Table 1. Parasite sampling.** Infections from each location were prescreened to remove multiple clone infections, and to include only a single representative of each multilocus genotype. These data illustrate the difference in parasite population structure from each location, with multiple clone infections common and identical multilocus genotypes rare in high transmission African populations, and multiple infections rare but identical multilocus genotypes common in S. American populations. | | | | | | | |
| --- | --- | --- | --- | --- | --- | --- | --- |
| Country^#^ | Location | Map Reference | Collection dates | Infections sampled | Single clone | Unique genotypes  (% of single clone) | Included in study |
| Africa |  |  |  |  |  |  |  |
| Malawi^1^ | Blantyre | -15° 47' 23.3", +35° 00' 34.6" | 2008 | 79 | 17 (22) | 17 (100) | 15 |
| The Gambia^2^ | Fajara | +13° 28' 23.7", -16° 41' 20.8" | 2008 | 188 | 49 (26) | ND* | 25 |
|  |  |  |  |  |  |  |  |
| Asia |  |  |  |  |  |  |  |
| Thailand^1^ | Mawker-Thai | +16° 20' 49.76", +98° 40' 5.15" | 2003-6 | 343 | 283(83) | 235(83) | 25 |
| Laos^1^ | Phalanxay | +16° 30' 39.3", +105° 30' 49.8" | 2008-10 | 93 | 58(62) | 50(86) | 28 |
| Cambodia^3^ | Pailin | +12° 51' 10.3", +102° 36' 51.0" | 2007-8 | 62 | 51 (82) | 32 (63) | 15 |
|  |  |  |  |  |  |  |  |
| South America |  |  |  |  |  |  |  |
| Venezuela^4^ | Las Claritas | +6° 10' 52.8", -61° 25' 17.6" | 2011-12 | 41 | 39(95) | 12(28) | 12 |
| Peru^4^ | Zungarochoca | -3°49'42.7"S +73°21'16.9"W |  | 40 | 30(75) | 6(20) | 2 |
|  |  |  |  |  |  |  |  |

^#^The genotyping methods used for prescreening varied between locations: ^1^ seven microsatellite loci [81], ^2^ two antigen loci (Merozoite surface antigen 1 and 2) [83], ^3^ 18 microsatellite loci [47], ^4^ 93 SNP loci [82]. *We were unable to determine unique genotypes from msp1/2 genotyping alone. However, following inspection of the 10,271 SNPs genotyped in Gambian isolates all parasites included were found to be unique.
